# A General Method for Computing the Homfly Polynomial of DNA Double Crossover 3-Regular Links

**DOI:** 10.1371/journal.pone.0125184

**Published:** 2015-05-01

**Authors:** Meilian Li, Qingying Deng, Xian’an Jin

**Affiliations:** 1 School of Mathematical Sciences, Xiamen University, Xiamen, Fujian, P. R. China; 2 School of Mathematics and Computer Sciences, Longyan University, Longyan, Fujian, P. R. China; Nankai University, CHINA

## Abstract

In the last 20 years or so, chemists and molecular biologists have synthesized some novel DNA polyhedra. Polyhedral links were introduced to model DNA polyhedra and study topological properties of DNA polyhedra. As a very powerful invariant of oriented links, the Homfly polynomial of some of such polyhedral links with small number of crossings has been obtained. However, it is a challenge to compute Homfly polynomials of polyhedral links with large number of crossings such as double crossover 3-regular links considered here. In this paper, a general method is given for computing the chain polynomial of the truncated cubic graph with two different labels from the chain polynomial of the original labeled cubic graph by substitutions. As a result, we can obtain the Homfly polynomial of the double crossover 3-regular link which has relatively large number of crossings.

## Introduction

**Fig 1:**
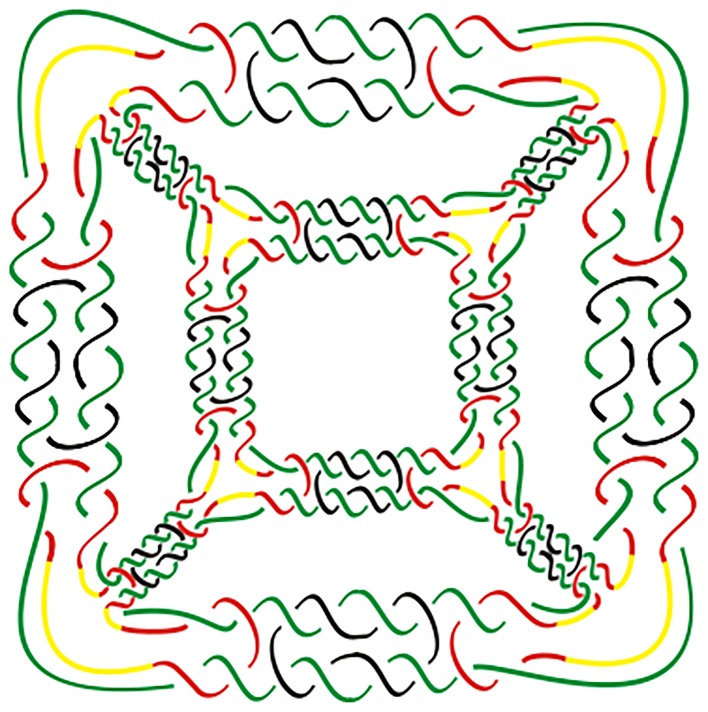
The planar graph of a double crossover hexahedral link with 16 × 12 = 192 crossings.

In the last 20 years or so, many DNA biomolecules with the shape of polyhedron have been synthesized by chemists and molecular biologists in the laboratory. For example, the DNA cube [1], DNA tetrahedron [2], DNA octahedron [3], DNA truncated octahedron [4], DNA bipyramid [5] and DNA dodecahedron [6]. In recent several years, a type of more complicated DNA polyhedra have been reported in [7–10]. They are all synthesized by the strategy of “*n*-point stars”. In fact they are called double crossover DNA polyhedra in [11]. In addition, similar DNA molecular structures can also be found in [12, 13]. Polyhedral links modelling the double crossover DNA polyhedra are called double crossover polyhedral links. As an example, the planar diagram of the double crossover hexahedral link is given in Fig 1.

**Fig 2:**
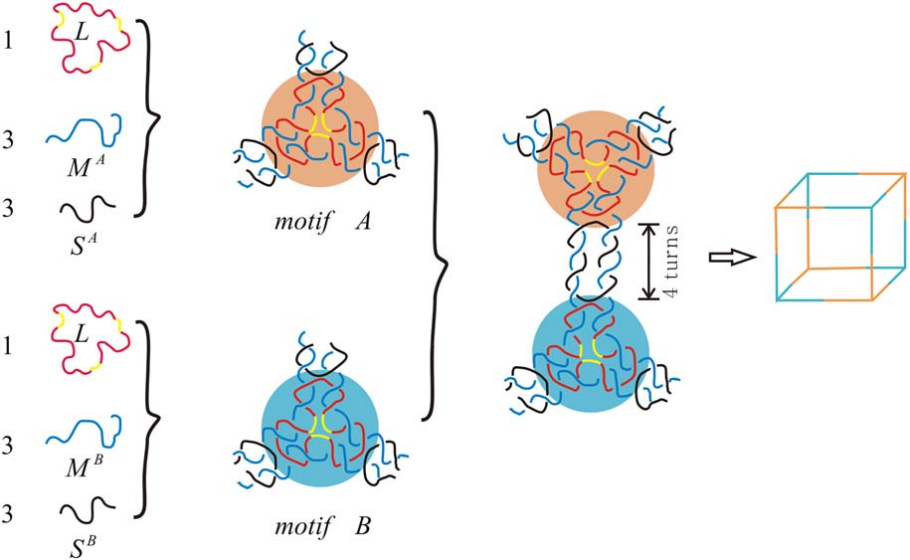
Assembly of DNA 4-turn hexahedra from two different component three-point-star tiles (A and B).

**Fig 3:**
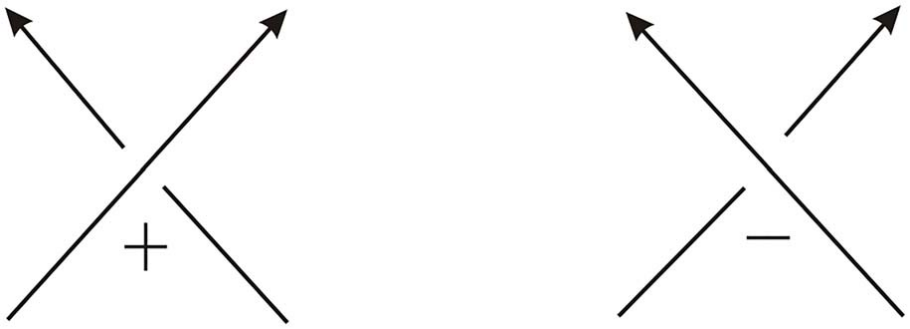
Right-handed (+) and left-handed crossings (−).

The DNA double crossover hexahedron was assembled from two different component three-point-star tiles (A and B), the process is shown in Fig 2. The hexahedral structures have been confirmed by multiple techniques including polyacrylamide gel electrophoresis (PAGE), dynamic light scattering (DLS), cryogenic electron microscopy (cryo-EM) imaging, and single particle three-dimensional (3D) reconstruction [9]. We shall use the orientation of the 2 backbone strands of the dsDNA to orient DNA polyhedral links. Thus we always consider DNA polyhedral links as oriented links with antiparallel orientations. Under this orientation, the double crossover hexahedral link in Fig 1 is a negative one, i.e., each crossing is left-handed. See Fig 3.

For understanding, describing and quantizing DNA polyhedra, many invariants of polyhedral links modeling DNA polyhedra have been computed and analyzed [14–26]. Among these invariants, the Homfly polynomial [27, 28] is a very powerful one. It bears much information of oriented links, containing the Jones polynomial [29] and Alexander-Conway polynomial [30, 31] as special cases. The Homfly polynomial can distinguish most links from their mirror images, and it helps to determine other numerical invariants such as braid index and the genus etc [32–34]. Unfortunately, computing the Homfly polynomial is, in general, very hard. Computer software (e.g. KnotGTK) can only deal with links with small (about 50) number of crossings.

**Fig 4:**
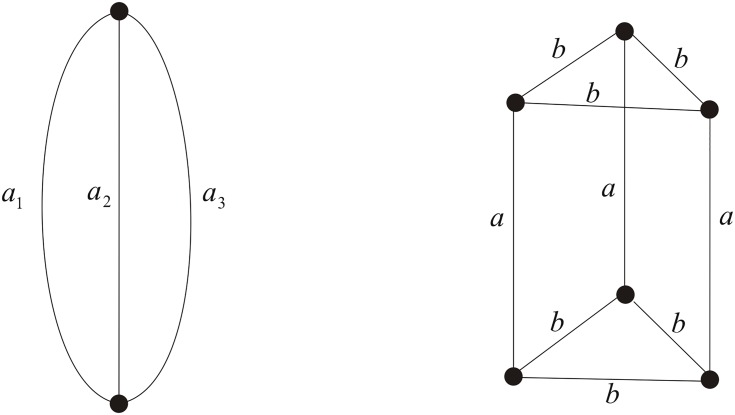
The labeled theta graph Θ and the labeled triangular prism truncated from the labeled theta graph Θ′.

**Fig 5:**
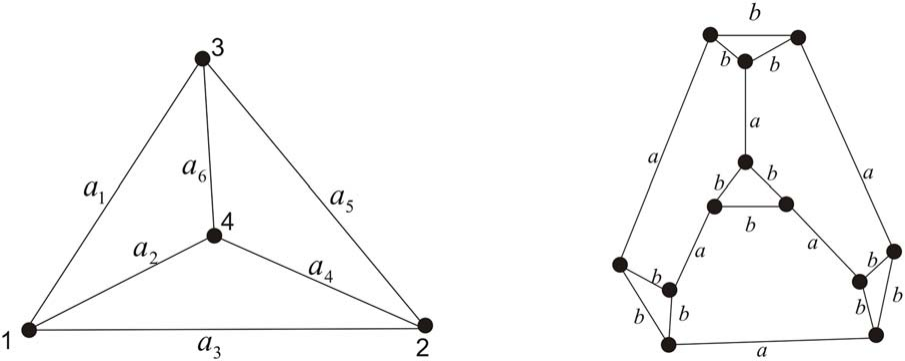
The labeled tetrahedral graph *T* and its truncation *T*′.

**Fig 6:**
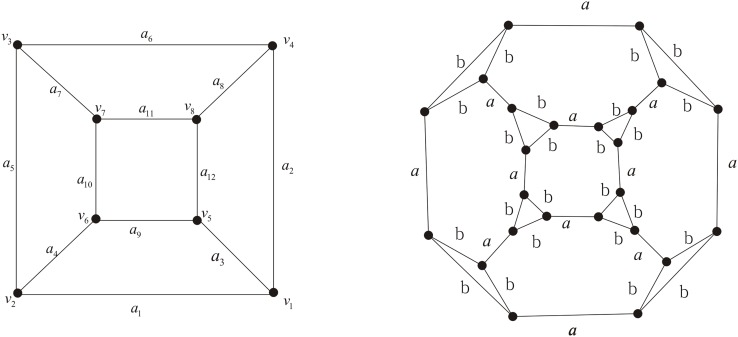
The labeled hexahedral graph *H* and its truncation.

Mathematically, given any planar (not necessarily polyhedral) graph, we can construct the corresponding double crossover link by covering the vertex of degree *n* with the *n*-point star. In this paper we shall focus on 3-regular, i.e. cubic plane graphs and call the corresponding double crossover links the double crossover 3-regular links. Based on results in [35] and [36], Cheng, Lei and Yang established a relation in [22] between the Homfly polynomial of the double crossover link and the chain polynomial [37] of the truncated graph with two distinct labels (See Figs 4–6 for examples). Using this relation, they obtained the Homfly polynomial of the double crossover tetrahedral link which has 96 crossings. To compute the Homfly polynomial of the double crossover 3-regular link with more large number of crossings, in the paper we give a general method to obtain the chain polynomial of a truncated cubic (i.e. 3-regular) graph with two different labels via the chain polynomial of the original cubic graph based on the *Y* − △ transform theorem in [39]. As a consequence, for example, we obtain the Homfly polynomial of the double crossover hexahedral link with 192 crossings.

## Method

We rely on two relationships in order to obtain the Homfly polynomial of double crossover 3-regular links. One relation (RI) is between the chain polynomial of a cubic labelled plane graph and that of its truncation with two different labels. See Theorem 1. The other (RII) is between the chain polynomial of truncated cubic graphs with two different labels and the Homfly polynomial of double crossover 3-regular links. See Theorem 2.

### 1. RI

The chain polynomial was introduced by Read and Whitehead in [37] for studying the chromatic polynomial of homeomorphic class of graphs. A chain in *G* is a path in which all vertices, except possibly the end vertices, have degree 2 in the graph *G*. The length of a chain will be the number of edges in it. A graph with edges labeled elements *a*, *b*, *c*, ⋯ of a commutative ring with unity 1 is called a labeled graph. Let *G* be a labeled graph. We usually identify the edges with their labels for convenience.

**Definition 1** The chain polynomial *Ch*(*G*) = *Ch*(*G*; *ω*; *a*, *b*, *c*, ⋯) of a labeled graph *G* is defined as

$$\begin{array}{c} Ch\left(G\right)=\sum_{Y}F_{Y}\pi_{U}, \end{array}$$

where the summation is over all subsets *Y* of the edge set *E* of the graph *G*; *F*_*Y*_ = *F*_*Y*_(1 − *ω*) denotes the flow polynomial in variable *ω* of ⟨*Y*⟩, the spanning subgraph of *G* with edge set *Y*; *π*_*U*_ denotes the product of the labels of the edges in *U* = *E* − *Y*.

For a survey on the flow polynomial of graphs, see [38].

**Proposition 1 ([37])** Let *G* be a labeled graph. Then

(1) If *G* has no edges, then *Ch*(*G*) = 1.

(2) If *G* consists of two graphs *A* and *B* having at most one vertex in common, then *Ch*(*G*) = *Ch*(*A*)*Ch*(*B*).

(3) The chain polynomial of a loop with the label *a* is *a* − *ω*.

(4) The term independent of the variables *a*, *b*, *c*, ⋯ is the flow polynomial of *G*.

(5) If *a* is an edge of *G* and is not a loop, let *H* be the graph obtained from *G* by deleting the edge *a*, and let *K* be the graph obtained by contracting it. Then

(i) *Ch*(*G*) = (*a* − 1)*Ch*(*H*) + *Ch*(*K*).

(ii) *Ch*(*H*) is the coefficient of *a* in *Ch*(*G*).

(iii) *Ch*(*K*) is obtained from *Ch*(*G*) by putting *a* = 1.

Since the flow polynomial of a graph with bridges is 0, we have:

**Lemma 1** Let *a*_1_, *a*_2_, ⋯, *a*_*s*_ be a chain of length *s* of a labeled graph *G*. Let *H* be the labeled graph obtained from *G* by replacing the chain *a*_1_, *a*_2_, ⋯, *a*_*s*_ by a single edge *a*. Then *Ch*(*H*) can be obtained from *Ch*(*G*) by replacing *a*_1_ *a*_2_ ⋯ *a*_*s*_ by *a* and conversely, *Ch*(*G*) can be obtained from *Ch*(*H*) by replacing *a* by *a*_1_ *a*_2_ ⋯ *a*_*s*_.

**Proposition 2 ([39])** Let *C* be a cut-set of edges in a graph *G*. Then any term in *Ch*(*G*) that contains the labels of all but one of the edges in *C* has zero coefficient.

**Fig 7:**
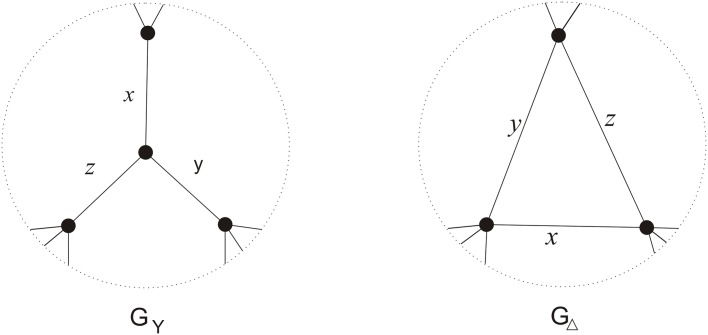
The *Y* − △ transformation.

In the case of the graph *G*_*Y*_ shown in Fig 7 (left), {*x*, *y*, *z*} is a cut-set. By Proposition 2, in *Ch*(*G*_*Y*_) there are no terms containing labels *xy* except *z*, *yz* except *x* or *xz* except *y*.

**Lemma 2 ([39], The *Y* − △ theorem)** Let *G*_*Y*_ be a labeled graph containing a vertex of degree 3 with incident edges labelled *x*, *y* and *z*. Let its chain polynomial be *Pxyz* + *Ax* + *By* + *Cz* + *V*. Let *G*_△_ be the labeled graph obtained from *G*_*Y*_ by a *Y* − △ transformation, where the rest of *G*_△_ is the same as in *G*_*Y*_, as shown in Fig 7. Then

$$\begin{array}{ccc} Ch(G_{\Delta }) & = & P(xyz-\omega )+A(yz+x-\omega -1)+B(xz+y-\omega -1)+\\ & & C(xy+z-\omega -1)+V(x+y+z-\omega -2). \end{array}$$

Lemma 2 implies that *Ch*(*G*_△_) can be obtained from *Ch*(*G*_*Y*_) = *Pxyz* + *Ax* + *By* + *Cz* + *V* by the following substitutions:

$$\begin{array}{ccc} xyz & ⟶ & xyz-\omega ,\\ x & ⟶ & yz+x-\omega -1,\\ y & ⟶ & xz+y-\omega -1,\\ z & ⟶ & xy+z-\omega -1,\\ V & ⟶ & V(x+y+z-\omega -2). \end{array}$$

Let *G* be a cubic graph, i.e. a 3-regular graph. By truncating *G* we mean inserting two vertices to each edge of *G* firstly, then doing the *Y* − △ transformation to each vertex of degree 3. Let *G*′ be the truncated graph of *G* with original edges of *G* labeled with *a* and newly produced edges labeled with *b*. See Figs 4–6 (right). Now we shall provide a general theorem to obtaining *Ch*(*G*′) via *Ch*(*G*) by substitutions.

**Theorem 1** Let *G* be a cubic graph with *n* vertices *v*_1_, *v*_2_, ⋯, *v*_*n*_ and *m* edges labeled *a*_1_, *a*_2_, ⋯, *a*_*m*_. Let *G*′ be the truncated graph of *G* with original edges of *G* labeled with *a* and newly produced edges labeled with *b*. Suppose

$$\begin{array}{c} Ch\left(G\right)=a_{1}a_{2}\cdots a_{m}+\sum_{i=1}^{m-1}\sum_{U_{ij}}F_{Y_{ij}}\pi_{U_{ij}}+F_{G}, \end{array}$$ (1)

where *U*_*ij*_ is a subset of cardinality *i* of {*a*_1_, *a*_2_, ⋯, *a*_*m*_} and $j=1,2,\cdots ,\left(\begin{array}{c} m\\ i \end{array}\right)$; *Y*_*ij*_ denotes the complementary subset of *U*_*ij*_. Then we can obtain *Ch*(*G*′), namely

$$\begin{array}{ccc} Ch(G^{{}^{\prime }}) & = & a^{m}{\left(b^{3}-\omega \right)}^{n}\\ & & +{\left(3b-\omega -2\right)}^{n}\sum_{i=1}^{m-1}a^{i}\sum_{U_{ij}}F_{Y_{ij}}{\left(\frac{b^{3}-\omega }{3b-\omega -2}\right)}^{p_{ij}}{\left(\frac{b^{2}+b-\omega -1}{3b-\omega -2}\right)}^{q_{ij}}\\ & & +F_{G}{\left(3b-\omega -2\right)}^{n}, \end{array}$$ (2)

where *p*_*ij*_ and *q*_*ij*_ are the numbers of *k*’s such that *q*_*k*_ = 3 and *q*_*k*_ = 1 in Eq (3).

**Proof.** We divide the whole proof into three steps.

**Fig 8:**
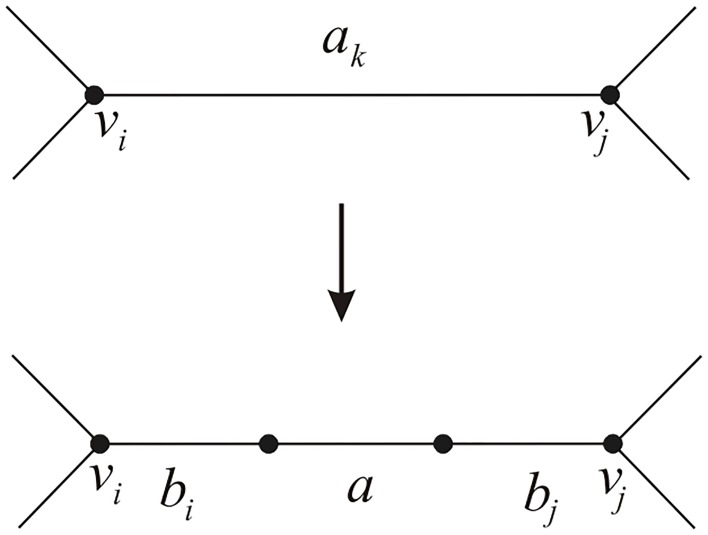
The construction of the labeled graph G* from the labeled graph *G*.

**Step 1.** Compute the chain polynomial of the labeled graph G* obtained from the labeled graph *G* by the replacements shown in Fig 8.

For each *k* = 1, 2, ⋯, *m*, we suppose that *v*_*i*_ and *v*_*j*_ are the end-vertices of the edge labeled *a*_*k*_. Recall that

$$\begin{array}{ccc} Ch(G) & = & \sum_{i=0}^{m}\sum_{U_{ij}}F_{Y_{ij}}\pi_{U_{ij}}\\ & = & a_{1}a_{2}\cdots a_{m}+\sum_{i=1}^{m-1}\sum_{U_{ij}}F_{Y_{ij}}\pi_{U_{ij}}+F_{G}, \end{array}$$

where *U*_*ij*_ is a subset of cardinality *i* of {*a*_1_, *a*_2_, ⋯, *a*_*m*_} and $j=1,2,\cdots ,\left(\begin{array}{c} m\\ i \end{array}\right)$; *Y*_*ij*_ denotes the complementary subset of *U*_*ij*_. By Lemma 1, replace *a*_*k*_ by *b*_*i*_ *ab*_*j*_ in *Ch*(*G*), we obtain *Ch*(G*). For a fixed *i* from 0 to *m*, $\sum_{U_{ij}}F_{Y_{ij}}\pi_{U_{ij}}$ becomes $a^{i}\sum_{U_{ij}}F_{Y_{ij}}\prod_{k=1}^{n}b_{k}^{q_{k}}$, where *q*_*k*_ is the number of edges in *U*_*ij*_ incident with the vertex *v*_*k*_. Thus we have:

$$\begin{array}{ccc} Ch(G*) & = & \sum_{i=0}^{m}a^{i}\sum_{U_{ij}}F_{Y_{ij}}\prod_{k=1}^{n}b_{k}^{q_{k}}\\ & = & a^{m}\prod_{i=1}^{n}b_{i}^{3}+\sum_{i=1}^{m-1}a^{i}\sum_{U_{ij}}F_{Y_{ij}}\prod_{k=1}^{n}b_{k}^{q_{k}}+F_{G}, \end{array}$$ (3)

where *q*_*k*_ = 0, 1, 2, 3 depending on *U*_*ij*_. Additionally, for nonzero terms in *Ch*(G*), *q*_*k*_ can not be 2. When *q*_*l*_ = 0, it means that *b*_*l*_ doesn’t appear in $\prod_{k=1}^{n}b_{k}^{q_{k}}$.

In the following, we apply Lemma 2 to each vertex of degree 3 of G*. Namely, for each *k* = 1, 2, ⋯, *n*, for each term of *Ch*(G*), replacing $b_{k}^{3}$ (namely *q*_*k*_ = 3) by $b_{k}^{3}-\omega$ and *b*_*k*_ by $b_{k}^{2}+b_{k}-\omega -1$, multiplying the other terms (namely, the term *V* in Lemma 2) by 3*b*_*k*_ − *ω* − 2. We divide it into two steps for clarity.

**Step 2.** For each *k* = 1, 2, ⋯, *n*, for each term of *Ch*(G*), replacing $b_{k}^{3}$ by *x* and *b*_*k*_ by *y*, we obtain a polynomial in *x*, *y*, *a*, *ω*, denoted it by *Ch*(G**), namely,

$$\begin{array}{c} Ch\left(G**\right)=a^{m}x^{n}+\sum_{i=1}^{m-1}a^{i}\sum_{U_{ij}}F_{Y_{ij}}x^{p_{ij}}y^{q_{ij}}+F_{G}, \end{array}$$ (4)

where *p*_*ij*_ and *q*_*ij*_ are the numbers of *k*’s such that *q*_*k*_ = 3 and *q*_*k*_ = 1 in *Ch*(G*), respectively. Note that *p*_*m*1_ = *n*, *q*_*m*1_ = 0, *p*_01_ = 0 and *q*_01_ = 0.

**Step 3.** In *Ch*(G**), replace *x* by $\frac{b^{3}-\omega }{3b-\omega -2}$ and *y* by $\frac{b^{2}+b-\omega -1}{3b-\omega -2}$, and normalize entire polynomial by (3*b* − *ω* − 2)^*n*^.

Therefore, the first term $a^{m}\prod_{i=1}^{n}b_{i}^{3}$ becomes *a* ^*m*^(*b* ^3^ − *ω*)^*n*^ and the last term *F*_*G*_ becomes *F*_*G*_(3*b* − *ω* − 2)^*n*^. Note that the numbers of *k*’s such that *q*_*k*_ = 0 is *n* − *p*_*ij*_ − *q*_*ij*_, which is exactly the times we need multiply the term corresponding to *U*_*ij*_ by 3*b* − *ω* − 2.

A polyhedral graph is planar, it is worth pointing out that our Theorem 1 applies to any cubic graphs which are not necessarily planar. Now we provide several examples.

**Example 1** The chain polynomial of the labeled theta graph Θ as shown in Fig 4 (left) and the more general labeled generalized theta *S*_*m*_ graph with *m* > 3 edges are given in [39] and [40]. Note that the triangular prism as shown in Fig 4 (right) is the truncated graph of the theta graph. Applying Theorem 1, we have

$$\begin{array}{c} Ch\left(\Theta \right)=a_{1}a_{2}a_{3}-\omega \left(a_{1}+a_{2}+a_{3}\right)+\omega^{2}+\omega . \end{array}$$

Step 1. *a*_1_ → *b*_1_ *ab*_2_, *a*_2_ → *b*_1_ *ab*_2_, *a*_3_ → *b*_1_ *ab*_2_.

$$\begin{array}{c} Ch\left(\Theta *\right)=a^{3}b_{1}^{3}b_{2}^{3}-3\omega ab_{1}b_{2}+\omega^{2}+\omega . \end{array}$$

Step 2. $b_{1}^{3}\to x,b_{2}^{3}\to x,b_{1}\to y,b_{2}\to y$

$$\begin{array}{c} Ch\left(\Theta^{**}\right)=a^{3}x^{2}-3\omega ay^{2}+\omega^{2}+\omega . \end{array}$$

Step 3. *x* → *b* ^3^ − *w*, *y* → *b* ^2^ + *b* − *w* − 1, then only need to multiply the last term by (3*b* − *ω* − 2)^2^, we obtain:

$$\begin{array}{c} Ch\left(\Theta \prime \right)=a^{3}{\left(b^{3}-w\right)}^{2}-3\omega a{\left(b^{2}+b-w-1\right)}^{2}+\left(\omega^{2}+\omega \right){\left(3b-\omega -2\right)}^{2}, \end{array}$$

which matches the result in [39].

**Example 2** Let *T* be the tetrahedral graph labeled as shown in Fig 5 (left), whose chain polynomial was calculated in [37, 39]. Applying Theorem 1, we have

$$\begin{array}{ccc} Ch(T) & = & a_{1}a_{2}a_{3}a_{4}a_{5}a_{6}-(a_{1}a_{2}a_{3}+a_{1}a_{5}a_{6}+a_{2}a_{4}a_{6}\\ & & +a_{3}a_{4}a_{5}+a_{1}a_{4}+a_{2}a_{5}+a_{3}a_{6})\omega +(a_{1}+a_{2}+a_{3}+a_{4}\\ & & +a_{5}+a_{6})\omega \left(\omega +1\right)-\omega \left(\omega +1\right)\left(\omega +2\right). \end{array}$$

Step 1. *a*_1_ → *b*_1_ *ab*_3_, *a*_2_ → *b*_1_ *ab*_4_, *a*_3_ → *b*_1_ *ab*_2_, *a*_4_ → *b*_2_ *ab*_4_, *a*_5_ → *b*_2_ *ab*_3_, *a*_6_ → *b*_3_ *ab*_4_.

$$\begin{array}{ccc} Ch(T*) & = & a^{6}b_{1}^{3}b_{2}^{3}b_{3}^{3}b_{4}^{3}-\omega a^{3}\left(b_{1}^{3}b_{2}b_{3}b_{4}+b_{1}b_{2}b_{3}^{3}b_{4}+b_{1}b_{2}b_{3}b_{4}^{3}+b_{1}b_{2}^{3}b_{3}b_{4}\right)\\ & & -\omega a^{2}\left(b_{1}b_{2}b_{3}b_{4}+b_{1}b_{2}b_{3}b_{4}+b_{1}b_{2}b_{3}b_{4}\right)+\omega \left(\omega +1\right)a(b_{1}b_{3}+b_{1}b_{4}\\ & & +b_{1}b_{2}+b_{2}b_{4}+b_{2}b_{3}+b_{3}b_{4})-\omega \left(\omega +1\right)\left(\omega +2\right). \end{array}$$

Step 2. $b_{k}^{3}\to x,b_{k}\to y,k=1,2,3,4.$

$$\begin{array}{c} Ch\left(T**\right)=a^{6}x^{4}-4\omega a^{3}xy^{3}-3\omega a^{2}y^{4}+6\omega \left(\omega +1\right)ay^{2}-\omega \left(\omega +1\right)\left(\omega +2\right). \end{array}$$

Step 3. *x* → *b* ^3^ − *w*, *y* → *b* ^2^ + *b* − *w* − 1, then multiplying every term by (3*b* − *ω* − 2)^4−*d*(*x*)−*d*(*y*)^, where *d*(*x*) and *d*(*y*) are degrees of *x* and *y* in the corresponding term in *Ch*(G**), respectively, we obtain:

$$\begin{array}{ccc} Ch(T\prime ) & = & a^{6}{\left(b^{3}-w\right)}^{4}-4\omega a^{3}\left(b^{3}-w\right){\left(b^{2}+b-w-1\right)}^{3}-3\omega a^{2}{\left(b^{2}+b-w-1\right)}^{4}\\ & & +6\omega \left(\omega +1\right)a{\left(b^{2}+b-w-1\right)}^{2}{\left(3b-\omega -2\right)}^{2}-\omega \left(\omega +1\right)\left(\omega +2\right){\left(3b-\omega -2\right)}^{4}, \end{array}$$

which matches the reuslt in [22].

**Example 3** Let *H* be the labeled hexahedral graph with *V*(*H*) = {*v*_1_, *v*_2_, ⋯, *v*_8_} and *E*(*H*) = {*a*_1_, *a*_2_, ⋯, *a*_12_} as shown in Fig 6 (left). By performing the Maple program in the Appendix of [22]) which can be used to compute the chain polynomial of labelled graph with small number of edges, we obtain the chain polynomial of the labeled hexahedral graph as follows.

$$\begin{array}{ccc} Ch(H) & = & -3\;a_{1}a_{7}w^{2}-a_{6}a_{3}a_{4}a_{10}a_{12}a_{9}w-a_{2}a_{6}a_{4}a_{12}a_{8}a_{11}w-a_{2}a_{6}a_{9}a_{10}a_{8}a_{4}w-\ldots \\ & & -a_{7}a_{10}w^{3}a_{11}-a_{12}a_{8}a_{11}w^{3}+a_{2}a_{4}a_{12}w^{2}+a_{2}a_{4}a_{12}w+a_{2}a_{9}w^{2}a_{11}+\ldots \\ & & -3\;a_{2}a_{7}w^{2}-2\;a_{2}a_{7}w-2\;a_{2}a_{10}w^{2}-a_{2}a_{10}w-a_{2}a_{12}w-3\;a_{2}a_{4}w^{2}-2\;a_{2}a_{4}w. \end{array}$$

According to Theorem 1, a simple program in the Maple platform for calculating *Ch*(*G*′) from *Ch*(*G*) can be written. See S1 Appendix. By applying the program, we obtain the chain polynomial of the truncated hexahedral graph *H*′ with two labels as shown in Fig 6 (right). Namely,

$$\begin{array}{ccc} Ch(H\prime ) & = & a^{12}{\left(b^{3}-\omega \right)}^{8}-\left(11\omega +25\omega^{2}+20\omega^{3}+7\omega^{4}+\omega^{5}\right){\left(3b-\omega -2\right)}^{8}\\ & & +\left(96\omega^{2}+48\omega +12\omega^{4}+60\omega^{3}\right){\left(b^{2}+b-\omega -1\right)}^{2}{\left(3b-\omega -2\right)}^{6}a\\ & & -\left(108\omega^{2}+66\omega +42\omega^{3}\right){\left(b^{2}+b-\omega -1\right)}^{4}{\left(3b-\omega -2\right)}^{4}a^{2}\\ & & -\left(24\omega^{2}+16\omega +8\omega^{3}\right)\left(b^{3}-\omega \right){\left(b^{2}+b-\omega -1\right)}^{3}{\left(3b-\omega -2\right)}^{4}a^{3}\\ & & +32\left(\omega^{2}+\omega \right){\left(b^{2}+b-\omega -1\right)}^{6}{\left(3b-\omega -2\right)}^{2}a^{3}\\ & & +24\left(\omega^{2}+\omega \right)\left(b^{3}-\omega \right){\left(b^{2}+b-\omega -1\right)}^{5}{\left(3b-\omega -2\right)}^{2}a^{4}\\ & & -6\omega {\left(b^{2}+b-\omega -1\right)}^{8}a^{4}+3\omega^{2}{\left(b^{2}+b-\omega -1\right)}^{8}a^{4}\\ & & +12\left(\omega^{2}+\omega \right){\left(b^{3}-\omega \right)}^{2}{\left(b^{2}+b-\omega -1\right)}^{4}{\left(3b-\omega -2\right)}^{2}a^{5}\\ & & -16\omega {\left(b^{3}-\omega \right)}^{2}{\left(b^{2}+b-\omega -1\right)}^{6}a^{6}\\ & & -6\omega {\left(b^{3}-\omega \right)}^{4}{\left(b^{2}+b-\omega -1\right)}^{4}a^{8}. \end{array}$$

### 2. RII

**Fig 9:**
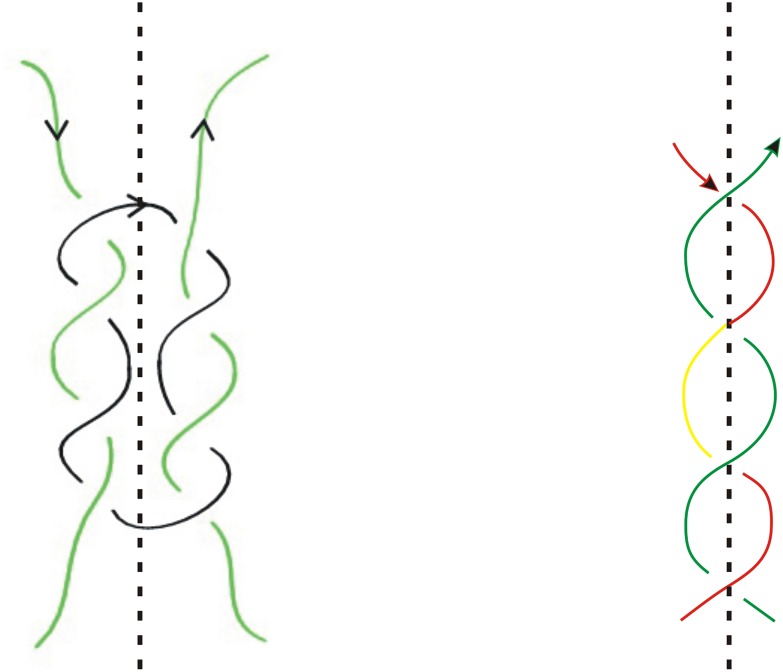
The double crossover tangle *T*_1_ (left) and the vertical integer tangle *T*_2_ (right).

In [22], a relation between the Homfly polynomial of positive double crossover polyhedral link and the chain polynomial of truncated polyhedral graph with two labels is obtained. For completeness we give an outline of the proof of the relation. Here we consider the negative double crossover polyhedral links and the two tangles *T*_1_ which is used to cover the original edge (labelled *a*) of a polyhedron and *T*_2_ which is used to cover the newly produced edge (labelled *b*) after truncation are shown in Fig 9.

Let *T* be a 2-tangle. We denote by *Nu*(*T*) and *De*(*T*) the numerator and denominator of the 2-tangle *T*, respectively. Let $\delta =\frac{v^{-1}-v}{z}$. After calculation, we obtain:

$$\begin{array}{ccc} P_{Nu(T_{1})}\left(v,z\right) & = & \delta^{-1}{\left(1-v^{-4}\right)}^{2}+\delta^{-1}2v^{-4}\left(1-v^{-4}\right)+\delta v^{-8}\\ P_{De(T_{1})}\left(v,z\right) & = & \delta^{-2}{\left(1-v^{-4}\right)}^{2}+2v^{-4}\left(1-v^{-4}\right)+\delta^{2}v^{-8}\\ P_{Nu(T_{2})}\left(v,z\right) & = & 1\\ P_{De(T_{2})}\left(v,z\right) & = & \delta^{-1}\left(1-v^{-4}\right)+\delta v^{-4}. \end{array}$$

Hence,

$$\begin{array}{ccc} \mu (T_{1}) & = & \frac{\delta P_{Nu(T_{1})}-P_{De(T_{1})}}{\delta^{2}-1}\\ & = & z^{2}{\left(v^{-1}+v^{-3}\right)}^{2}\\ w(T_{1}) & = & \frac{\delta P_{De(T_{1})}-P_{Nu(T_{1})}}{\delta P_{Nu(T_{1})}-P_{De(T_{1})}}\\ & = & \frac{v^{-3}-v^{-1}-2z^{2}\left(v+v^{-1}\right)}{z^{3}{\left(1+v^{2}\right)}^{2}}\\ \mu (T_{2}) & = & \frac{\delta P_{Nu(T_{2})}-P_{De(T_{2})}}{\delta^{2}-1}\\ & = & -z(v^{-1}+v^{-3})\\ w(T_{2}) & = & \frac{\delta P_{De(T_{2})}-P_{Nu(T_{2})}}{\delta P_{Nu(T_{2})}-P_{De(T_{2})}}\\ & = & -z^{-1}{\left(v+v^{3}\right)}^{-1}. \end{array}$$

After changing *v* to −*v* ^−1^, you will find *μ*(*T*_1_), *μ*(*T*_2_) and *w*(*T*_1_), *w*(*T*_2_) coincide with *μ*_*x*_(*e*), *μ*_*y*_(*e*) and *w*_*x*_(*e*), *w*_*y*_(*e*) in Theorem 3.3 of [22].

Let *P* be a polyhedral graph. Let *P*′ be the truncated polyhedral graph of *P*. Let *L*(*P*) be the negative double crossover 4-turn link based on *P*. Then [35]

$$\begin{array}{c} P_{L(P)}\left(v,z\right)=\delta^{-1}{\left[z\left(v^{-1}+v^{-3}\right)\right]}^{4|E(P)|}Q_{P^{\prime }}\left(\delta ,\delta \right), \end{array}$$

where the weights of the original edges and the newly produced edges by truncation are $\frac{v^{-3}-v^{-1}-2z^{2}(v+v^{-1})}{z^{3}{\left(1+v^{2}\right)}^{2}}$ and −*z* ^−1^(*v* + *v* ^3^)^−1^, respectively. Combining it with the relation between the dichromatic polynomial *Q*_*P*′_ and the chain polynomial *Ch*(*P*′) obtained in [36] (see Lemma 2.5), we obtain:

**Theorem 2** Let *P* be a polyhedral graph having *x* edges. Let*P*′ be the truncated polyhedral graph of *P* with two labels *a* and *b* (having 2*x* vertices and 3*x* edges). Let *L*(*P*) be the negative double crossover 4-turn link based on *P*. In *Ch*(*P*′), we let

$$\begin{array}{ccc} w & = & 1-\delta^{2},\\ a & = & \frac{v^{-2}-1-z^{2}\left(1+v^{2}+v^{4}+v^{6}\right)}{v^{-2}-1-2z^{2}\left(v^{2}+1\right)},\\ b & = & v^{4}. \end{array}$$

Then

$$\begin{array}{c} P_{L(P)}\left(v,z\right)=\delta^{-1}{\left[\frac{v^{-3}-v^{-1}-2z^{2}\left(v+v^{-1}\right)}{v^{13}-v^{15}}\right]}^{x}Ch(P^{’}). \end{array}$$

After changing *v* to −*v* ^−1^, you can find Theorem 2 coincides with Theorem 3.4 in [22].

## Results

In this section we use Theorem 2 to compute the Homfly polynomial of negative double crossover 3-regular links based on the theta graph, the tetrahedron and the cube. To obtain the Homfly polynomial of the positive double crossover polyhedral links, one only need change *v* to −*v* ^−1^. Recall that the Conway and Jones polynomials are both special cases of the Homfly polynomial, i.e.

$$\begin{array}{ccc} & & \nabla_{L}\left(z\right)=P_{L}\left(1,z\right),\\ & & V_{L}\left(t\right)=P_{L}\left(t,\sqrt{t}-\frac{1}{\sqrt{t}}\right). \end{array}$$

Some computational results are too long to be included in this paper, we only list a few terms here:

$$\begin{array}{ccc} P_{L(\Theta )}\left(v,z\right) & = & -v^{-55}z^{-7}(v^{48}z^{14}+7\;v^{46}z^{14}+28\;v^{44}z^{14}+84\;v^{42}z^{14}+3\;v^{42}z^{12}+207\;v^{40}z^{14}\\ & & +15\;v^{40}z^{12}+441\;v^{38}z^{14}+47\;v^{38}z^{12}+838\;v^{36}z^{14}+115\;v^{36}z^{12}+1450\;v^{34}z^{14}\\ & & +3\;v^{36}z^{10}+246\;v^{34}z^{12}+2308\;v^{32}z^{14}+9\;v^{34}z^{10}+478\;v^{32}z^{12}+3388\;v^{30}z^{14}\\ & & +36\;v^{32}z^{10}+836\;v^{30}z^{12}+4570\;v^{28}z^{14}+84\;v^{30}z^{10}+1332\;v^{28}z^{12}+5590\;v^{26}z^{14}\\ & & +4\;v^{30}z^{8}+196\;v^{28}z^{10}+1834\;v^{26}z^{12}+6069\;v^{24}z^{14}+4\;v^{28}z^{8}+372\;v^{26}z^{10}\\ & & +2066\;v^{24}z^{12}+5619\;v^{22}z^{14}+52\;v^{26}z^{8}+528\;v^{24}z^{10}+1554\;v^{22}z^{12}+4116\;v^{20}z^{14}\\ & & +52\;v^{24}z^{8}+664\;v^{22}z^{10}-374\;v^{20}z^{12}+2140\;v^{18}z^{14}+9\;v^{24}z^{6}+238\;v^{22}z^{8}\\ & & -285\;v^{20}z^{10}-2815\;v^{18}z^{12}+679\;v^{16}z^{14}-9\;v^{22}z^{6}+238\;v^{20}z^{8}-2319\;v^{18}z^{10}\\ & & -3291\;v^{16}z^{12}+97\;v^{14}z^{14}+184\;v^{20}z^{6}-1066\;v^{18}z^{8}-1996\;v^{16}z^{10}-1705\;v^{14}z^{12}\\ & & -184\;v^{18}z^{6}-1066\;v^{16}z^{8}+684\;v^{14}z^{10}-341\;v^{12}z^{12}+66\;v^{18}z^{4}-606\;v^{16}z^{6}\\ & & +1190\;v^{14}z^{8}+1518\;v^{12}z^{10}-198\;v^{16}z^{4}+606\;v^{14}z^{6}+1190\;v^{12}z^{8}+506\;v^{10}z^{10}\\ & & +12\;v^{16}z^{2}+66\;v^{14}z^{4}+624\;v^{12}z^{6}-418\;v^{10}z^{8}-60\;v^{14}z^{2}+330\;v^{12}z^{4}\\ & & -624\;v^{10}z^{6}-418\;v^{8}z^{8}+v^{14}+108\;v^{12}z^{2}-330\;v^{10}z^{4}-211\;v^{8}z^{6}-7\;v^{12}\\ & & -60\;v^{10}z^{2}-66\;v^{8}z^{4}+211\;v^{6}z^{6}+21\;v^{10}-60\;v^{8}z^{2}+198\;v^{6}z^{4}-35\;v^{8}\\ & & +108\;v^{6}z^{2}-66\;v^{4}z^{4}+35\;v^{6}-60\;v^{4}z^{2}-21\;v^{4}+12\;v^{2}z^{2}+7\;v^{2}-1), \end{array}$$

$$\begin{array}{ccc} P_{L(T)}\left(v,z\right) & = & -v^{-109}z^{-13}(v^{96}z^{26}+13\;v^{94}z^{26}+91\;v^{92}z^{26}+455\;v^{90}z^{26}+6\;v^{90}z^{24}\\ & & +1814\;v^{88}z^{26}+66\;v^{88}z^{24}+6110\;v^{86}z^{26}+400\;v^{86}z^{24}+18014\;v^{84}z^{26}\\ & & +15\;v^{84}z^{22}+6240\;v^{82}z^{24}+\cdots +76044\;v^{44}z^{16}-568318\;v^{42}z^{18}\\ & & -9151952\;v^{40}z^{20}-35714154\;v^{38}z^{22}-\cdots -3982176\;v^{26}z^{14}+2351088\;v^{24}z^{16}\\ & & +3395394\;v^{22}z^{18}-464792\;v^{20}z^{20}+\cdots +24\;v^{2}z^{2}+13\;v^{2}-1), \end{array}$$

$$\begin{array}{ccc} P_{L(H)}\left(v,z\right) & = & -v^{-217}z^{-25}(v^{192}z^{50}+25\;v^{190}z^{50}+325\;v^{188}z^{50}+2925\;v^{186}z^{50}+12\;v^{186}z^{48}\\ & & +20463\;v^{184}z^{50}+276\;v^{184}z^{48}+118455\;v^{182}z^{50}+3320\;v^{182}z^{48}+589867\;v^{180}z^{50}\\ & & +27784\;v^{180}z^{48}+2594275\;v^{178}z^{50}+\cdots +117286813536\;v^{146}z^{44}+\cdots \\ & & +24631386348\;v^{118}z^{36}+\cdots -4342184782394\;v^{82}z^{34}-\cdots \\ & & +604406814588216v^{62}z^{36}+\cdots -316550564\;v^{42}z^{6}+\cdots \\ & & +6981260760v^{24}z^{10}-\cdots +48\;v^{2}z^{2}+25\;v^{2}-1). \end{array}$$

Thus,

$$\begin{array}{ccc} \nabla_{L(\Theta )}\left(z\right) & = & -37632\;z^{7},\\ \nabla_{L(T)}\left(z\right) & = & -1078984704\;z^{13},\\ \nabla_{L(H)}\left(z\right) & = & -748419423085264896\;z^{25}, \end{array}$$

$$\begin{array}{ccc} V_{L(\Theta )}\left(z\right) & = & -t^{\frac{-103}{2}}(t^{48}-7\;t^{47}+28\;t^{46}-84\;t^{45}+210\;t^{44}-462\;t^{43}+924\;t^{42}-1713\;t^{41}\\ & & +2985\;t^{40}-4939\;t^{39}+7819\;t^{38}-11912\;t^{37}+17544\;t^{36}-25072\;t^{35}+34875\;t^{34}\\ & & -47326\;t^{33}+62766\;t^{32}-81462\;t^{31}+103570\;t^{30}-129055\;t^{29}+157634\;t^{28}\\ & & -188690\;t^{27}+221242\;t^{26}-253870\;t^{25}+284755\;t^{24}-311685\;t^{23}+332298\;t^{22}\\ & & -344228\;t^{21}+345601\;t^{20}-335293\;t^{19}+313457\;t^{18}-281464\;t^{17}+242045\;t^{16}\\ & & -198659\;t^{15}+155160\;t^{14}-114883\;t^{13}+80386\;t^{12}-52914\;t^{11}+32652\;t^{10}\\ & & -18771\;t^{9}+10012\;t^{8}-4907\;t^{7}+2200\;t^{6}-885\;t^{5}+320\;t^{4}-98\;t^{3}+27\;t^{2}\\ & & -5\;t+1), \end{array}$$

$$\begin{array}{ccc} V_{L(T)}\left(z\right) & = & -t^{\frac{-205}{2}}(t^{96}-13\;t^{95}+91\;t^{94}-455\;t^{93}+1820\;t^{92}-6188\;t^{91}+18564\;t^{90}\\ & & -50382\;t^{89}+125898\;t^{88}-\cdots +405708071163\;t^{42}-422901756090\;t^{41})\\ & & +434762332438\;t^{40}-440420890844\;t^{39}+\cdots +90t^{2}-11t+1), \end{array}$$

$$\begin{array}{ccc} V_{L(H)}\left(z\right) & = & -t^{\frac{-409}{2}}(t^{192}-25\;t^{191}+325\;t^{190}-2925\;t^{189}+20475\;t^{188}\\ & & -118755\;t^{187}+593775\;t^{186}-\cdots +64833446416942962011t^{136}-\cdots \\ & & -451043083493105466629441t^{89}+\cdots -388424807064142369273t^{39}+\cdots \\ & & +1083862665t^{10}-\cdots +324t^{2}-23t+1). \end{array}$$

To our surprise their Conway polynomials are so simple, all having only one term. Let *L* = *K*_1_ ∪ *K*_2_ ∪ ⋯ ∪ *K*_*n*_ be an oriented link with *n* components. Let *A*(*L*) = (*a*_*ij*_)_*n*×*n*_ be the linking matrix with *a*_*ij*_ = *lk*(*K*_*i*_, *K*_*j*_), the linking number of *K*_*i*_ and *K*_*j*_, if *i* ≠ *j* and $a_{ii}=-\sum_{j=1,j\neq i}^{n}lk(K_{i},K_{j})$. In [41, 42] the authors proved that the first coefficient of ∇_*L*_(*z*) (i.e. the coefficient of the term of the lowest degree *n* − 1) is equal to the cofactor of *A*(*L*) up to a sign. Our computational results are the same to the results obtained from cofactor of linking matrices.

Let *P*_*L*_(*v*, *z*) be the Homfly polynomial of the oriented link *L*. In [43] and [44], Franks, Williams and Morton independently gave a lower bound for the braid index *b*(*L*) of an oriented link *L* in terms of *span*_*v*_ *P*_*L*_(*v*, *z*) as follows:

$$\begin{array}{c} \frac{1}{2}\;span_{v}P_{L}\left(v,z\right)+1\leq b\left(L\right), \end{array}$$ (5)

where *span*_*v*_ *P*_*L*_(*v*, *z*) = max deg_*v*_ *P*_*L*_(*v*, *z*) − min deg_*v*_ *P*_*L*_(*v*, *z*), and max deg_*v*_ *P*_*L*_(*v*, *z*) and min deg_*v*_ *P*_*L*_(*v*, *z*) denote, respectively, the maximal degree and minimal degree of *v* in the polynomial *P*_*L*_(*v*, *z*). This inequality (5) is usually called MFW inequality. By combining the following result obtained by Ohyama in 1993 [45] which states that for a non-splittable oriented link *L*,

$$\begin{array}{c} b\left(L\right)\leq 1+\frac{c(L)}{2}, \end{array}$$ (6)

where *c*(*L*) is the crossing number of *L*.

Hence, *b*(*L*(*θ*)) = 25, *b*(*L*(*T*)) = 49 and *b*(*L*(*H*)) = 97. These results coincide with results in [21].

## Discussion

A general method is given in this paper for computing the chain polynomial of the truncated cubic graph with two different labels from the chain polynomial of the original labeled cubic graph by substitutions. Hence, we convert the computation of the chain polynomial of a large graph with two labels to that of the chain polynomial of a small graph. As an application, by combining with the relation between the Homfly polynomial of a double crossover polyhedral link and the chain polynomial of the truncated polyhedral graph with two different labels, we obtain the Homfly polynomial of the double crossover hexahedral link, which has 192 crossings.

From our computational results, we know that the double crossover DNA hexahedral link is topologically chiral and its braid index is 97. More deeply chemical and biological understanding of our computational results deserves further exploring. To our surprise the Conway polynomials of the double crossover links based on the theta graph, the tetrahedral graph and the hexahedral graph all have only one term. It may coincide with Corollary 4.6 in [35].

We only consider double crossover 3-regular links. Similar approach may be developed to deal with double crossover *n*-regular links. It may be a more difficult task to compute the Homfly polynomial of polyhedral link modeling protein polyhedra in [46, 47].

## Supporting Information

S1 Appendix file details the computational result for the Homfly polynomial of the positive double crossover 4-turn hexahedral link. (PDF)
