# Supplementary material for: A General Method for Computing the Homfly Polynomial of DNA Double Crossover 3-Regular Links
Source: PLoS One. 2015 May 1;10(5):e0125184. doi: 10.1371/journal.pone.0125184 (PMC4416910; doi:10.1371/journal.pone.0125184)
Supplement: S1 Appendix — (PDF) [file pone.0125184.s001.pdf]

$$-v^{25}z^{-25}(v^{192}-48v^{190}z^{2}+1128v^{188}z^{4}-17260v^{186}z^{6}+192972v^{184}z^{8}-1677140v^{182}z^{10}+$$
$$11770606v^{180}z^{12}-68410605v^{178}z^{14}+335020933v^{176}z^{16}-1399222238v^{174}z^{18}+$$
$$5025169250v^{172}z^{20}-15601034121v^{170}z^{22}+41986355772v^{168}z^{24}-98012016076v^{166}z^{26}+$$
$$198174275724v^{164}z^{28}-345878757392v^{162}z^{30}+518221017686v^{160}z^{32}-661326077972v^{158}z^{34}+$$
$$711223343664v^{156}z^{36}-635463637048v^{154}z^{38}+462667279152v^{152}z^{40}-267177081683v^{150}z^{42}+$$
$$117599632268v^{148}z^{44}-37024081218v^{146}z^{46}+7417923052v^{144}z^{48}-709969407v^{142}z^{50}-$$
$$25v^{190}+1104v^{188}z^{2}-23688v^{186}z^{4}+327940v^{184}z^{6}-3280524v^{182}z^{8}+$$
$$25157100v^{180}z^{10}-153017878v^{178}z^{12}+752516655v^{176}z^{14}-3015188397v^{174}z^{16}+$$
$$9794555666v^{172}z^{18}-25125846250v^{170}z^{20}+46803102363v^{168}z^{22}-41986355772v^{166}z^{24}-$$
$$98012016076v^{164}z^{26}+594522827172v^{162}z^{28}-1729393786960v^{160}z^{30}+3627547123802v^{158}z^{32}-$$
$$5951934701748v^{156}z^{34}+7823456780304v^{154}z^{36}-8261027281624v^{152}z^{38}+6940009187280v^{150}z^{40}-$$
$$4542010388611v^{148}z^{42}+2234393013092v^{146}z^{44}-777505705578v^{144}z^{46}+170612230196v^{142}z^{48}-$$
$$17749235175v^{140}z^{50}+300v^{188}-12096v^{186}z^{2}+234624v^{184}z^{4}-2899716v^{182}z^{6}+$$
$$25473864v^{180}z^{8}-167746980v^{178}z^{10}+847935516v^{176}z^{12}-3288207363v^{174}z^{14}+$$
$$9415158252v^{172}z^{16}-17003483622v^{170}z^{18}+1074295508v^{168}z^{20}+120292339677v^{166}z^{22}-$$
$$487874158992v^{164}z^{24}+1128442069052v^{162}z^{26}-1464607617000v^{160}z^{28}-258952171296v^{158}z^{30}+$$
$$6687119240792v^{156}z^{32}-19227968684796v^{154}z^{34}+35035231091496v^{152}z^{36}-46681173785176v^{150}z^{38}+$$
$$47040512676384v^{148}z^{40}-35774187972699v^{146}z^{42}+20007549417320v^{144}z^{44}-7789107385230v^{142}z^{46}+$$
$$1888877391984v^{140}z^{48}-215053531323v^{138}z^{50}-2300v^{186}+83904v^{184}z^{2}-1452864v^{182}z^{4}+$$
$$15741804v^{180}z^{6}-118125384v^{178}z^{8}+637807900v^{176}z^{10}-2454160540v^{174}z^{12}+$$
$$6069614793v^{172}z^{14}-4331400348v^{170}z^{16}-37688505302v^{168}z^{18}+195635292460v^{166}z^{20}-$$
$$485685291999v^{164}z^{22}+487874158992v^{162}z^{24}+1128442069052v^{160}z^{26}-5979217056792v^{158}z^{28}+$$
$$12540389439200v^{156}z^{30}-11230919295288v^{154}z^{32}-14333459449884v^{152}z^{34}+72449270794296v^{150}z^{36}-$$
$$144237731436344v^{148}z^{38}+187420337495520v^{146}z^{40}-172128198229227v^{144}z^{42}+$$
$$112016277358040v^{142}z^{44}-49537084938390v^{140}z^{46}+13416427501136v^{138}z^{48}-1684497366675v^{136}z^{50}+$$
$$12650v^{184}-412896v^{182}z^{2}+6278448v^{180}z^{4}-58103208v^{178}z^{6}+$$
$$355660356v^{176}z^{8}-1428867600v^{174}z^{10}+3116449090v^{172}z^{12}+2109809958v^{170}z^{14}-$$
$$43922165598v^{168}z^{16}+162531754826v^{166}z^{18}-251103340130v^{164}z^{20}-307953946620v^{162}z^{22}+$$
$$2583453828540v^{160}z^{24}-5911022316808v^{158}z^{26}+3439306470360v^{156}z^{28}+17175054092548v^{154}z^{30}-$$
$$55272068575764v^{152}z^{32}+68257188714420v^{150}z^{34}+19518971696940v^{148}z^{36}-234084149949332v^{146}z^{38}+$$
$$471393548919312v^{144}z^{40}-559589082107319v^{142}z^{42}+438279174964880v^{140}z^{44}-$$
$$224517852947784v^{138}z^{46}+68766868338412v^{136}z^{48}-9603212730129v^{134}z^{50}-53130v^{182}+$$

$1530144\,v^{180}z^{2} - 19976880\,v^{178}z^{4} + 151541112\,v^{176}z^{6} - 689864964\,v^{174}z^{8}+$

$1485070320\,v^{172}z^{10} + 2660139222\,v^{170}z^{12} - 31207304898\,v^{168}z^{14} + 98057710206\,v^{166}z^{16}-$

$64567526982\,v^{164}z^{18} - 590975117030\,v^{162}z^{20} + 2260625376180\,v^{160}z^{22} - 2583453828540\,v^{158}z^{24}-$

$5911022316808\,v^{156}z^{26} + 26790962964456\,v^{154}z^{28} - 33817055464812\,v^{152}z^{30} - 33087509363244\,v^{150}z^{32}+$

$181786555698516\,v^{148}z^{34} - 242343627650652\,v^{146}z^{36} - 54573794386436\,v^{144}z^{38}+$

$711478363171056\,v^{142}z^{40} - 1273334314645095\,v^{140}z^{42} + 1266777486758672\,v^{138}z^{44}-$

$771631278018216\,v^{136}z^{46} + 271318069590900\,v^{134}z^{48} - 42547079923785\,v^{132}z^{50}+$

$177100\,v^{180} - 4420416\,v^{178}z^{2} + 47944512\,v^{176}z^{4} - 276557336\,v^{174}z^{6}+$

$710696400\,v^{172}z^{8} + 1280076200\,v^{170}z^{10} - 16641892936\,v^{168}z^{12} + 49944763626\,v^{166}z^{14}+$

$4821411364\,v^{164}z^{16} - 484478328886\,v^{162}z^{18} + 1353350015568\,v^{160}z^{20} - 92610770156\,v^{158}z^{22}-$

$8229531936344\,v^{156}z^{24} + 18586543507800\,v^{154}z^{26} + 2305097282008\,v^{152}z^{28} - 83942057524940\,v^{150}z^{30}+$

$136541681373680\,v^{148}z^{32} + 63706876631692\,v^{146}z^{34} - 515796293272136\,v^{144}z^{36} + 604296437448708\,v^{142}z^{38}$

$+ 372179242470904\,v^{140}z^{40} - 1986549240295647\,v^{138}z^{42} + 2778805844748768\,v^{136}z^{44}-$

$2088548842830048\,v^{134}z^{46} + 858625401689368\,v^{132}z^{48} - 152818768901589\,v^{130}z^{50} - 480700\,v^{178}+$

$10152384\,v^{176}z^{2} - 86756736\,v^{174}z^{4} + 317761928\,v^{172}z^{6} + 243743280\,v^{170}z^{8}-$

$6981260760\,v^{168}z^{10} + 23361404872\,v^{166}z^{12} + 9982451490\,v^{164}z^{14} - 283346783604\,v^{162}z^{16}+$

$667663124122\,v^{160}z^{18} + 674783735952\,v^{158}z^{20} - 6196089871452\,v^{156}z^{22} + 8229531936344\,v^{154}z^{24}+$

$18586543507800\,v^{152}z^{26} - 70018290576984\,v^{150}z^{28} + 27249908581604\,v^{148}z^{30} + 224744136634928\,v^{146}z^{32}-$

$377903650909140\,v^{144}z^{34} - 225945302150392\,v^{142}z^{36} + 1301317293023124\,v^{140}z^{38}-$

$962057892807288\,v^{138}z^{40} - 1808148830892399\,v^{136}z^{42} + 4645633979804160\,v^{134}z^{44}-$

$4561385118483936\,v^{132}z^{46} + 2243169260059304\,v^{130}z^{48} - 458529699905565\,v^{128}z^{50}+$

$1081575\,v^{176} - 18689616\,v^{174}z^{2} + 113868216\,v^{172}z^{4} - 97974756\,v^{170}z^{6}-$

$2205136020\,v^{168}z^{8} + 10062897300\,v^{166}z^{10} + 1373145870\,v^{164}z^{12} - 131554983135\,v^{162}z^{14}+$

$308504980035\,v^{160}z^{16} + 542244874290\,v^{158}z^{18} - 3551206277310\,v^{156}z^{20} + 2556300208518\,v^{154}z^{22}+$

$17519928506796\,v^{152}z^{24} - 38946054239838\,v^{150}z^{26} - 31799586246636\,v^{148}z^{28}+$

$198020985204732\,v^{146}z^{30} - 86890102432972\,v^{144}z^{32} - 573006029020746\,v^{142}z^{34}+$

$745311394091220\,v^{140}z^{36} + 967155673207560\,v^{138}z^{38} - 2681151828611034\,v^{136}z^{40}+$

$129749371160997\,v^{134}z^{42} + 5745221237051832\,v^{132}z^{44} - 8159362553561760\,v^{130}z^{46}+$

$4943289963518064\,v^{128}z^{48} - 1175922466798830\,v^{126}z^{50} - 2042975\,v^{174} + 27457584\,v^{172}z^{2}-$

$92179032\,v^{170}z^{4} - 428611348\,v^{168}z^{6} + 3679440084\,v^{166}z^{8} - 2845910700\,v^{164}z^{10}-$

$48528452310\,v^{162}z^{12} + 141406130613\,v^{160}z^{14} + 249555821045\,v^{158}z^{16}-$

$1732378601822\,v^{156}z^{18} + 680672796630\,v^{154}z^{20} + 11011979758958\,v^{152}z^{22}-$

$17519928506796\,v^{150}z^{24} - 38946054239838\,v^{148}z^{26} + 116961210273052\,v^{146}z^{28}+$

$64445596374828\,v^{144}z^{30} - 466245796355188\,v^{142}z^{32} + 58864375983718\,v^{140}z^{34} +$

$1348500680454268\,v^{138}z^{36} - 762343451898392\,v^{136}z^{38} - 3109181164474054\,v^{134}z^{40} +$

$3502296968582261\,v^{132}z^{42} + 4597853209754248\,v^{130}z^{44} - 12031172224657056\,v^{128}z^{46} +$

$9345974581576208\,v^{126}z^{48} - 2625521071518270\,v^{124}z^{50} + 3268760\,v^{172} - 31380096\,v^{170}z^{2} +$

$980993332\,v^{166}z^{6} - 2511027144\,v^{164}z^{8} - 12803335980\,v^{162}z^{10} + 60886915812\,v^{160}z^{12} +$

$65733009279\,v^{158}z^{14} - 742741184040\,v^{156}z^{16} + 320880923762\,v^{154}z^{18} +$

$5492139461172\,v^{152}z^{20} - 7494939115254\,v^{150}z^{22} - 26161003588392\,v^{148}z^{24} +$

$57007310543778\,v^{146}z^{26} + 84983995068672\,v^{144}z^{28} - 264668875315788\,v^{142}z^{30} -$

$212956954848064\,v^{140}z^{32} + 891080050232598\,v^{138}z^{34} + 503086549946288\,v^{136}z^{36} -$

$2473827698265920\,v^{134}z^{38} - 1121741874649488\,v^{132}z^{40} + 6367794150853861\,v^{130}z^{42} +$

$533851272411904\,v^{128}z^{44} - 14547800155210056\,v^{126}z^{46} + 15369232605998608\,v^{124}z^{48} -$

$5183309149374582\,v^{122}z^{50} - 4457400\,v^{170} + 25674624\,v^{168}z^{2} + 134078592\,v^{166}z^{4} -$

$1071291516\,v^{164}z^{6} - 1466174520\,v^{162}z^{8} + 21612878100\,v^{160}z^{10} - 1868435556\,v^{158}z^{12} -$

$269824587165\,v^{156}z^{14} + 254357427144\,v^{154}z^{16} + 2250490068162\,v^{152}z^{18} - 3482631194388\,v^{150}z^{20} -$

$13107422139630\,v^{148}z^{22} + 26161003588392\,v^{146}z^{24} + 57007310543778\,v^{144}z^{26} - 127731231859776\,v^{142}z^{28} -$

$207782088712860\,v^{140}z^{30} + 436980477960224\,v^{138}z^{32} + 707984230489638\,v^{136}z^{34} -$

$1101665270296560\,v^{134}z^{36} - 2272559211967616\,v^{132}z^{38} + 2223967986994512\,v^{130}z^{40} +$

$6486636836801493\,v^{128}z^{42} - 5366209136615264\,v^{126}z^{44} - 14007774338621544\,v^{124}z^{46} +$

$22231061033867376\,v^{122}z^{48} - 9170450180127270\,v^{120}z^{50} + 5200300\,v^{168} - 9984576\,v^{166}z^{2} -$

$234637536\,v^{164}z^{4} + 470344848\,v^{162}z^{6} + 5215423620\,v^{160}z^{8} - 11086394360\,v^{158}z^{10} -$

$73700497518\,v^{156}z^{12} + 167260719072\,v^{154}z^{14} + 719829778812\,v^{152}z^{16} -$

$1717910763958\,v^{150}z^{18} - 5104867560878\,v^{148}z^{20} + 12172278200700\,v^{146}z^{22} +$

$27923957395580\,v^{144}z^{24} - 59290135647034\,v^{142}z^{26} - 126815178192104\,v^{140}z^{28} +$

$192060402385284\,v^{138}z^{30} + 496970224180888\,v^{136}z^{32} - 344519405989454\,v^{134}z^{34} -$

$1636927728544680\,v^{132}z^{36} - 168813805782448\,v^{130}z^{38} + 4395339848304436\,v^{128}z^{40} +$

$3331316066575293\,v^{126}z^{42} - 10623266389373760\,v^{124}z^{44} - 9635373937112916\,v^{122}z^{46} +$

$28533462505557024\,v^{120}z^{48} - 14714221934568590\,v^{118}z^{50} - 5200300\,v^{166} - 9984576\,v^{164}z^{2} +$

$234637536\,v^{162}z^{4} + 466065744\,v^{160}z^{6} - 5247580740\,v^{158}z^{8} - 10409191800\,v^{156}z^{10} +$

$75976831590\,v^{154}z^{12} + 144940579488\,v^{152}z^{14} - 771775075836\,v^{150}z^{16} -$

$1408803251654\,v^{148}z^{18} + 5577097430710\,v^{146}z^{20} + 10300370932044\,v^{144}z^{22} -$

$27923957395580\,v^{142}z^{24} - 59290135647034\,v^{140}z^{26} + 87732156071688\,v^{138}z^{28} +$

$267227149227732\,v^{136}z^{30} - 94048441312376\,v^{134}z^{32} - 883521340019838\,v^{132}z^{34} -$

$604406814588216\,v^{130}z^{36} + 1851372402176720\,v^{128}z^{38} + 3866780637245388\,v^{126}z^{40} -$

$$1363054084004403\,v^{124}z^{42} - 12884478414451872\,v^{122}z^{44} - 2150541341177892\,v^{120}z^{46} +$$
$$32687246132849376\,v^{118}z^{48} - 21641265142334750\,v^{116}z^{50} + 4457400\,v^{164} +$$
$$25674624\,v^{162}z^{2} - 134078592\,v^{160}z^{4} - 1069419408\,v^{158}z^{6} + 1538089824\,v^{156}z^{8} +$$
$$21450946800\,v^{154}z^{10} - 2843328720\,v^{152}z^{12} - 268170759048\,v^{150}z^{14} -$$
$$151465554240\,v^{148}z^{16} + 2255010914994\,v^{146}z^{18} + 2445227273560\,v^{144}z^{20} -$$
$$12734000404404\,v^{142}z^{22} - 21241609577784\,v^{140}z^{24} + 43524091222118\,v^{138}z^{26} +$$
$$119550220121856\,v^{136}z^{28} - 41439997208052\,v^{134}z^{30} - 421081490240960\,v^{132}z^{32} -$$
$$396198537600678\,v^{130}z^{34} + 664674468763992\,v^{128}z^{36} + 2189814844532456\,v^{126}z^{38} +$$
$$1406095474179000\,v^{124}z^{40} - 5062926856919355\,v^{122}z^{42} - 11340521242690320\,v^{120}z^{44} +$$
$$6512257516878012\,v^{118}z^{46} + 33474878680288584\,v^{116}z^{48} - 29457779467114862\,v^{114}z^{50} -$$
$$3268760\,v^{162} - 31380096\,v^{160}z^{2} + 982173232\,v^{156}z^{6} + 2450779296\,v^{154}z^{8} -$$
$$13206488080\,v^{152}z^{10} - 57911993008\,v^{150}z^{12} + 84207448344\,v^{148}z^{14} +$$
$$694176058976\,v^{146}z^{16} + 5422172098\,v^{144}z^{18} - 5027418555352\,v^{142}z^{20} -$$
$$4871389783620\,v^{140}z^{22} + 21241609577784\,v^{138}z^{24} + 43524091222118\,v^{136}z^{26} -$$
$$31360986041600\,v^{134}z^{28} - 184625886216228\,v^{132}z^{30} - 168177626159584\,v^{130}z^{32} +$$
$$263439694241674\,v^{128}z^{34} + 979167302865544\,v^{126}z^{36} + 1118432214203400\,v^{124}z^{38} -$$
$$1066663304866040\,v^{122}z^{40} - 6290182097436555\,v^{120}z^{42} - 6957874862311312\,v^{118}z^{44} +$$
$$14104109275700172\,v^{116}z^{46} + 30464116637258616\,v^{114}z^{48} - 37431920175388830\,v^{112}z^{50} +$$
$$2042975\,v^{160} + 27457584\,v^{158}z^{2} + 92179032\,v^{156}z^{4} - 431506036\,v^{154}z^{6} -$$
$$3665048700\,v^{152}z^{8} - 2286355500\,v^{150}z^{10} + 49330833274\,v^{148}z^{12} +$$
$$122924451021\,v^{146}z^{14} - 296653338357\,v^{144}z^{16} - 1479522228802\,v^{142}z^{18} +$$
$$70931046734\,v^{140}z^{20} + 8829697771152\,v^{138}z^{22} + 11198942221806\,v^{136}z^{24} -$$
$$21761570633833\,v^{134}z^{26} - 71481712040532\,v^{132}z^{28} - 45201905952807\,v^{130}z^{30} +$$
$$126600667096058\,v^{128}z^{32} + 389351353032057\,v^{126}z^{34} + 501829115259188\,v^{124}z^{36} -$$
$$114649962019716\,v^{122}z^{38} - 2276102183852622\,v^{120}z^{40} - 5217649181982042\,v^{118}z^{42} -$$
$$1619561904519100\,v^{116}z^{44} + 18969340541667006\,v^{114}z^{46} + 24095855349787788\,v^{112}z^{48} -$$
$$44747401830309591\,v^{110}z^{50} - 1081575\,v^{158} - 18689616\,v^{156}z^{2} - 113868216\,v^{154}z^{4} -$$
$$95351652\,v^{152}z^{6} + 2230709820\,v^{150}z^{8} + 9778145940\,v^{148}z^{10} - 4253341938\,v^{146}z^{12} -$$
$$129419820111\,v^{144}z^{14} - 234092720835\,v^{142}z^{16} + 627336412878\,v^{140}z^{18} +$$
$$2686637991578\,v^{138}z^{20} + 859076441808\,v^{136}z^{22} - 11198942221806\,v^{134}z^{24} -$$
$$21761570633833\,v^{132}z^{26} - 811957874940\,v^{130}z^{28} + 61774016564349\,v^{128}z^{30} +$$
$$136332513752246\,v^{126}z^{32} + 159320748329409\,v^{124}z^{34} - 22123027495236\,v^{122}z^{36} -$$
$$726394333998132\,v^{120}z^{38} - 2177376544808274\,v^{118}z^{40} - 2987312273419770\,v^{116}z^{42} +$$
$$2960060478276524\,v^{114}z^{44} + 20494114802051478\,v^{112}z^{46} + 15463706035929108\,v^{110}z^{48}$$

$-50671517107584255\,v^{108}z^{50}+480700\,v^{156}+10152384\,v^{154}z^{2}+86756736\,v^{152}z^{4}+$
$316550564\,v^{150}z^{6}-278955720\,v^{148}z^{8}-7055955900\,v^{146}z^{10}-$
$21362290140\,v^{144}z^{12}+20366263371\,v^{142}z^{14}+263207354540\,v^{140}z^{16}+$
$436780624350\,v^{138}z^{18}-825164248460\,v^{136}z^{20}-3934154283928\,v^{134}z^{22}-$
$3811096910872\,v^{132}z^{24}+6756649750223\,v^{130}z^{26}+24809304532392\,v^{128}z^{28}+$
$37294029363767\,v^{126}z^{30}+30417094256240\,v^{124}z^{32}-29238617549763\,v^{122}z^{34}-$
$235572005412368\,v^{120}z^{36}-726781177827100\,v^{118}z^{38}-1399457693191824\,v^{116}z^{40}-$
$753579254484058\,v^{114}z^{42}+5850966671331256\,v^{112}z^{44}+19027364305974778\,v^{110}z^{46}+$
$5937678867027592\,v^{108}z^{48}-54684783349185803\,v^{106}z^{50}-177100\,v^{154}-4420416\,v^{152}z^{2}-$
$47944512\,v^{150}z^{4}-276639308\,v^{148}z^{6}-689793336\,v^{146}z^{8}+$
$1500903300\,v^{144}z^{10}+16609289820\,v^{142}z^{12}+41203418271\,v^{140}z^{14}-$
$35412742812\,v^{138}z^{16}-413722745586\,v^{136}z^{18}-758003637620\,v^{134}z^{20}+$
$395535755208\,v^{132}z^{22}+3811096910872\,v^{130}z^{24}+6756649750223\,v^{128}z^{26}+$
$5382075181464\,v^{126}z^{28}-1599644479637\,v^{124}z^{30}-20224846748656\,v^{122}z^{32}-$
$75282133378251\,v^{120}z^{34}-212195231113360\,v^{118}z^{36}-453704574162316\,v^{116}z^{38}-$
$557775599126256\,v^{114}z^{40}+828860534164678\,v^{112}z^{42}+6937397453573416\,v^{110}z^{44}+$
$15492809077332114\,v^{108}z^{46}-3196867961736712\,v^{106}z^{48}-56543700548533475\,v^{104}z^{50}+$
$53130\,v^{152}+1530144\,v^{150}z^{2}+19976880\,v^{148}z^{4}+152170392\,v^{146}z^{6}+$
$686665836\,v^{144}z^{8}+1333703520\,v^{142}z^{10}-3585645258\,v^{140}z^{12}-$
$30365296866\,v^{138}z^{14}-70472820990\,v^{136}z^{16}+17610799590\,v^{134}z^{18}+$
$455645315926\,v^{132}z^{20}+1001333851056\,v^{130}z^{22}+689540285976\,v^{128}z^{24}-$
$972016157017\,v^{126}z^{26}-3397979849580\,v^{124}z^{28}-7751080134582\,v^{122}z^{30}-$
$20383362615964\,v^{120}z^{32}-52818828535101\,v^{118}z^{34}-115183538980620\,v^{116}z^{36}-$
$176451149815120\,v^{114}z^{38}+33812360687628\,v^{112}z^{40}+1621997591699658\,v^{110}z^{42}+$
$6617210494990648\,v^{108}z^{44}+10960744976972868\,v^{106}z^{46}-10977408970609212\,v^{104}z^{48}-$
$56275994454107741\,v^{102}z^{50}-12650\,v^{150}-412896\,v^{148}z^{2}-6278448\,v^{146}z^{4}-$
$58673736\,v^{144}z^{6}-361039020\,v^{142}z^{8}-1396253600\,v^{140}z^{10}-2443751406\,v^{138}z^{12}+$
$5152705302\,v^{136}z^{14}+41816709342\,v^{134}z^{16}+97806442358\,v^{132}z^{18}+$
$49331276546\,v^{130}z^{20}-256454534864\,v^{128}z^{22}-689540285976\,v^{126}z^{24}-$
$972016157017\,v^{124}z^{26}-1530860560740\,v^{122}z^{28}-3890651013422\,v^{120}z^{30}-$
$10225537056836\,v^{118}z^{32}-22309352581253\,v^{116}z^{34}-35227158877284\,v^{114}z^{36}-$
$1441717542416\,v^{112}z^{38}+327267381232692\,v^{110}z^{40}+1790264318685930\,v^{108}z^{42}+$
$5459044786266344\,v^{106}z^{44}+6346006825664404\,v^{104}z^{46}-16854060194891044\,v^{102}z^{48}-$

$54126913052949045\,v^{100}z^{50} + 2300\,v^{148} + 83904\,v^{146}z^{2} + 1452864\,v^{144}z^{4} +$

$16057128\,v^{142}z^{6} + 123652560\,v^{140}z^{8} + 663761640\,v^{138}z^{10} + 2314229112\,v^{136}z^{12} +$

$3995694306\,v^{134}z^{14} - 3964722972\,v^{132}z^{16} - 39302894498\,v^{130}z^{18} -$

$91263238048\,v^{128}z^{20} - 90501362136\,v^{126}z^{22} - 16404433296\,v^{124}z^{24} -$

$43327744873\,v^{122}z^{26} - 429028413096\,v^{120}z^{28} - 1350673224490\,v^{118}z^{30} - 2818329607640\,v^{116}z^{32} -$

$3414912234993\,v^{114}z^{34} + 5957474426592\,v^{112}z^{36} + 72982921878808\,v^{110}z^{38} +$

$402981522180912\,v^{108}z^{40} + 1584869252712282\,v^{106}z^{42} + 3972159200483960\,v^{104}z^{44} +$

$2272324618079412\,v^{102}z^{46} - 20662359042461672\,v^{100}z^{48} - 50481865581592017\,v^{98}z^{50} -$

$300\,v^{146} - 12096\,v^{144}z^{2} - 234624\,v^{142}z^{4} - 3020856\,v^{140}z^{6} - 28389840\,v^{138}z^{8} -$

$195279000\,v^{136}z^{10} - 945014520\,v^{134}z^{12} - 2975421126\,v^{132}z^{14} -$

$4933389684\,v^{130}z^{16} + 877309134\,v^{128}z^{18} + 21732702080\,v^{126}z^{20} +$

$39533839944\,v^{124}z^{22} + 16404433296\,v^{122}z^{24} - 43327744873\,v^{120}z^{26} -$

$92483406648\,v^{118}z^{28} - 65405572098\,v^{116}z^{30} + 491965795672\,v^{114}z^{32} +$

$3653943959127\,v^{112}z^{34} + 18386571497184\,v^{110}z^{36} + 85752427819192\,v^{108}z^{38} +$

$361654826550480\,v^{106}z^{40} + 1219751165597370\,v^{104}z^{42} + 2511403829083880\,v^{102}z^{44} -$

$935044165140828\,v^{100}z^{46} - 22530883689743832\,v^{98}z^{48} - 45787670492044665\,v^{96}z^{50} +$

$25\,v^{144} + 1104\,v^{142}z^{2} + 23688\,v^{140}z^{4} + 360772\,v^{138}z^{6} +$

$4278084\,v^{136}z^{8} + 38216300\,v^{134}z^{10} + 243260810\,v^{132}z^{12} +$

$1043007735\,v^{130}z^{14} + 2773631261\,v^{128}z^{16} + 3612337414\,v^{126}z^{18} -$

$1191186570\,v^{124}z^{20} - 10574666062\,v^{122}z^{22} - 9214346262\,v^{120}z^{24} +$

$14660690671\,v^{118}z^{26} + 68042910264\,v^{116}z^{28} + 275942191365\,v^{114}z^{30} +$

$1138205560832\,v^{112}z^{32} + 4342184782394\,v^{110}z^{34} + 17323058006296\,v^{108}z^{36} +$

$72049979801636\,v^{106}z^{38} + 276602139106090\,v^{104}z^{40} + 833642949701376\,v^{102}z^{42} +$

$1273825975749640\,v^{100}z^{44} - 3188469890103548\,v^{98}z^{46} - 22769960567091784\,v^{96}z^{48} -$

$40486995299745858\,v^{94}z^{50} - v^{142} - 48\,v^{140}z^{2} - 1128\,v^{138}z^{4} - 23308\,v^{136}z^{6} -$

$420996\,v^{134}z^{8} - 5485140\,v^{132}z^{10} - 47921954\,v^{130}z^{12} -$

$271895421\,v^{128}z^{14} - 960396789\,v^{126}z^{16} - 1882225450\,v^{124}z^{18} -$

$1040406126\,v^{122}z^{20} + 3623840490\,v^{120}z^{22} + 9214346262\,v^{118}z^{24} +$

$14660690671\,v^{116}z^{26} + 52550537640\,v^{114}z^{28} + 229209365433\,v^{112}z^{30} +$

$828824500160\,v^{110}z^{32} + 3064672637706\,v^{108}z^{34} + 12612908072264\,v^{106}z^{36} +$

$51896955640340\,v^{104}z^{38} + 188124462622326\,v^{102}z^{40} + 498539633155584\,v^{100}z^{42} +$

$335528571490264\,v^{98}z^{44} - 4557233780134380\,v^{96}z^{46} - 21771351940842488\,v^{94}z^{48} -$

$34972511465127282\,v^{92}z^{50} + 684\,v^{134}z^{6} + 33864\,v^{132}z^{8} +$

$722700\,v^{130}z^{10} + 8888412\,v^{128}z^{12} + 66785433\,v^{126}z^{14} +$

$307422048\,v^{124}z^{16} + 829442982\,v^{122}z^{18} + 1099632108\,v^{120}z^{20}+$
$2194974\,v^{118}z^{22} - 1309223280\,v^{116}z^{24} + 4715770855\,v^{114}z^{26}+$
$33619991736\,v^{112}z^{28} + 118834070883\,v^{110}z^{30} + 425233693688\,v^{108}z^{32}+$
$1834236484602\,v^{106}z^{34} + 8197998488952\,v^{104}z^{36} + 33504172685588\,v^{102}z^{38}+$
$113705430303552\,v^{100}z^{40} + 241349429684928\,v^{98}z^{42} - 304639277108648\,v^{96}z^{44}-$
$5194372485228060\,v^{94}z^{46} - 19932719008684104\,v^{92}z^{48} - 29561158245761826\,v^{90}z^{50}-$
$36\,v^{132}z^{6} - 3144\,v^{130}z^{8} - 92980\,v^{128}z^{10} - 1550044\,v^{126}z^{12}-$
$15279339\,v^{124}z^{14} - 90043616\,v^{122}z^{16} - 312107242\,v^{120}z^{18}-$
$582505932\,v^{118}z^{20} - 303440330\,v^{116}z^{22} + 1309223280\,v^{114}z^{24} + 4715770855\,v^{112}z^{26}+$
$11251272552\,v^{110}z^{28} + 37853249735\,v^{108}z^{30} + 200188154504\,v^{106}z^{32} + 1055927082090\,v^{104}z^{34}+$
$4871992889128\,v^{102}z^{36} + 19258061917924\,v^{100}z^{38} + 58424840879104\,v^{98}z^{40}+$
$63057751122368\,v^{96}z^{42} - 688925801667704\,v^{94}z^{44} - 5281802402436780\,v^{92}z^{46}-$
$17609389288427896\,v^{90}z^{48} - 24485295764065458\,v^{88}z^{50} + 204\,v^{128}z^{8} + 9000\,v^{126}z^{10}+$
$223990\,v^{124}z^{12} + 3068604\,v^{122}z^{14} + 23719680\,v^{120}z^{16} + 105212110\,v^{118}z^{18}+$
$264128070\,v^{116}z^{20} + 357820092\,v^{114}z^{22} + 195729516\,v^{112}z^{24}-$
$250945457\,v^{110}z^{26} + 231552108\,v^{108}z^{28} + 13702157380\,v^{106}z^{30}+$
$105822351392\,v^{104}z^{32} + 569893590678\,v^{102}z^{34} + 2547720336656\,v^{100}z^{36}+$
$9353247130204\,v^{98}z^{38} + 21356345056272\,v^{96}z^{40} - 48023256721004\,v^{94}z^{42}-$
$875320030849232\,v^{92}z^{44} - 4994864968245540\,v^{90}z^{46} - 15089794819290896\,v^{88}z^{48}-$
$19896052740692514\,v^{86}z^{50} - 12\,v^{126}z^{8} - 600\,v^{124}z^{10} - 26190\,v^{122}z^{12}-$
$544404\,v^{120}z^{14} - 5705088\,v^{118}z^{16} - 32201442\,v^{116}z^{18} - 98684910\,v^{114}z^{20}-$
$165334260\,v^{112}z^{22} - 195729516\,v^{110}z^{24} - 250945457\,v^{108}z^{26} + 560830404\,v^{106}z^{28}+$
$8214362580\,v^{104}z^{30} + 51353588768\,v^{102}z^{32} + 255273967878\,v^{100}z^{34}+$
$1060837268592\,v^{98}z^{36} + 3156378479404\,v^{96}z^{38} - 988114294992\,v^{94}z^{40}-$
$107941124793900\,v^{92}z^{42} - 921919431910448\,v^{90}z^{44} - 4483159974806004\,v^{88}z^{46}-$
$12588521245576944\,v^{86}z^{48} - 15874146133696914\,v^{84}z^{50} + 2080\,v^{120}z^{12}+$
$83292\,v^{118}z^{14} + 1261752\,v^{116}z^{16} + 9323686\,v^{114}z^{18} + 34437752\,v^{112}z^{20}+$
$56450844\,v^{110}z^{22} + 21333256\,v^{108}z^{24} + 22208239\,v^{106}z^{26}+$
$489854408\,v^{104}z^{28} + 3088977796\,v^{102}z^{30} + 16234318184\,v^{100}z^{32}+$
$72267893150\,v^{98}z^{34} + 221645076128\,v^{96}z^{36} - 276146576116\,v^{94}z^{38}-$
$12716189632648\,v^{92}z^{40} - 132344078614884\,v^{90}z^{42} - 878781808002016\,v^{88}z^{44}-$
$3863214273204508\,v^{86}z^{46} - 10250739292304672\,v^{84}z^{48} - 12444213035409018\,v^{82}z^{50}-$
$160\,v^{118}z^{12} - 12372\,v^{116}z^{14} - 273144\,v^{114}z^{16} - 2767786\,v^{112}z^{18}-$
$13722808\,v^{110}z^{20} - 31169844\,v^{108}z^{22} - 21333256\,v^{106}z^{24}+$

$22208239\,v^{104}z^{26}+18624120\,v^{102}z^{28}-109340812\,v^{100}z^{30}-$
$1246618600\,v^{98}z^{32}-15662740050\,v^{96}z^{34}-178832114912\,v^{94}z^{36}-$
$1878953942404\,v^{92}z^{38}-17502752834232\,v^{90}z^{40}-134224129999908\,v^{88}z^{42}-$
$784919632580320\,v^{86}z^{44}-3218680367297580\,v^{84}z^{46}-8162770527337312\,v^{82}z^{48}-$
$9589758737884554\,v^{80}z^{50}+1320\,v^{114}z^{14}+47088\,v^{112}z^{16}+692328\,v^{110}z^{18}+$
$4722968\,v^{108}z^{20}+14551809\,v^{106}z^{22}+14408550\,v^{104}z^{24}-$
$29951615\,v^{102}z^{26}-202059876\,v^{100}z^{28}-1162518843\,v^{98}z^{30}-$
$7279106014\,v^{96}z^{32}-46774031628\,v^{94}z^{34}-321156784992\,v^{92}z^{36}-$
$2399699372704\,v^{90}z^{38}-18167023175076\,v^{88}z^{40}-123286983417582\,v^{86}z^{42}-$
$668381967147064\,v^{84}z^{44}-2604576996044628\,v^{82}z^{46}-6364936545631896\,v^{80}z^{48}-$
$7266875662132632\,v^{78}z^{50}-120\,v^{112}z^{14}-6512\,v^{110}z^{16}-147928\,v^{108}z^{18}-$
$1467800\,v^{106}z^{20}-6596739\,v^{104}z^{22}-14408550\,v^{102}z^{24}-29951615\,v^{100}z^{26}-$
$172197580\,v^{98}z^{28}-1199063655\,v^{96}z^{30}-7763230546\,v^{94}z^{32}-49762552300\,v^{92}z^{34}-$
$332690896608\,v^{90}z^{36}-2352706450144\,v^{88}z^{38}-16648360601948\,v^{86}z^{40}-106215355523790\,v^{84}z^{42}-$
$547851955294568\,v^{82}z^{44}-2053110601032292\,v^{80}z^{46}-4864212758159656\,v^{78}z^{48}-$
$5415760735429080\,v^{76}z^{50}+432\,v^{108}z^{16}+21616\,v^{106}z^{18}+342040\,v^{104}z^{20}+2019411\,v^{102}z^{22}+$
$3500856\,v^{100}z^{24}-12452159\,v^{98}z^{26}-130995936\,v^{96}z^{28}-920156901\,v^{94}z^{30}-$
$6225643120\,v^{92}z^{32}-41961612828\,v^{90}z^{34}-289641523248\,v^{88}z^{36}-2051813468440\,v^{86}z^{38}-$
$14171367472920\,v^{84}z^{40}-87289509535110\,v^{82}z^{42}-434724902390528\,v^{80}z^{44}-1579569824464764\,v^{78}z^{46}-$
$3645328714584872\,v^{76}z^{48}-3969695911382448\,v^{74}z^{50}-48\,v^{106}z^{16}-$
$3216\,v^{104}z^{18}-85592\,v^{102}z^{20}-819825\,v^{100}z^{22}-3500856\,v^{98}z^{24}-$
$12452159\,v^{96}z^{26}-78454944\,v^{94}z^{28}-599654529\,v^{92}z^{30}-4420807984\,v^{90}z^{32}-$
$31912797756\,v^{88}z^{34}-231251469264\,v^{86}z^{36}-1670208994552\,v^{84}z^{38}-11453930182824\,v^{82}z^{40}-$
$69045446309286\,v^{80}z^{42}-335098646908544\,v^{78}z^{44}-1187520427042572\,v^{76}z^{46}-$
$2679754591897752\,v^{74}z^{48}-2861567190478512\,v^{72}z^{50}+56\,v^{102}z^{18}+10740\,v^{100}z^{20}+$
$167380\,v^{98}z^{22}+545668\,v^{96}z^{24}-3005971\,v^{94}z^{26}-42580908\,v^{92}z^{28}-368530170\,v^{90}z^{30}-$
$2948752492\,v^{88}z^{32}-22911549100\,v^{86}z^{34}-174954773820\,v^{84}z^{36}-1294909104304\,v^{82}z^{38}-$
$8884703706280\,v^{80}z^{40}-52827957186498\,v^{78}z^{42}-251437518680984\,v^{76}z^{44}-873013035267688\,v^{74}z^{46}-$
$1932512476913088\,v^{72}z^{48}-2028224698780816\,v^{70}z^{50}-8\,v^{100}z^{18}-2148\,v^{98}z^{20}-$
$62076\,v^{96}z^{22}-545668\,v^{94}z^{24}-3005971\,v^{92}z^{26}-23373828\,v^{90}z^{28}-$
$214875138\,v^{88}z^{30}-1889317780\,v^{86}z^{32}-15822898188\,v^{84}z^{34}-127103705556\,v^{82}z^{36}-$
$964796491888\,v^{80}z^{38}-6649874210328\,v^{78}z^{40}-39205258585698\,v^{76}z^{42}-$

$183861371167688\,v^{74}z^{44} - 627789461669640\,v^{72}z^{46} - 1367020256653248\,v^{70}z^{48} -$

$1413082563402640\,v^{68}z^{50} + 7068\,v^{94}z^{22} + 59160\,v^{92}z^{24} - 563203\,v^{90}z^{26} -$

$11112528\,v^{88}z^{28} - 121715694\,v^{86}z^{30} - 1175963128\,v^{84}z^{32} - 10572588948\,v^{82}z^{34} -$

$89067118536\,v^{80}z^{36} - 693421465240\,v^{78}z^{38} - 4815288895488\,v^{76}z^{40} - 28262267995482\,v^{74}z^{42} -$

$131097322953416\,v^{72}z^{44} - 441600278559648\,v^{70}z^{46} - 948291497213112\,v^{68}z^{48} - 967370035381096\,v^{66}z^{50} -$

$2356\,v^{92}z^{22} - 59160\,v^{90}z^{24} - 563203\,v^{88}z^{26} - 5797008\,v^{86}z^{28} - 67013046\,v^{84}z^{30} -$

$709124008\,v^{82}z^{32} - 6827981044\,v^{80}z^{34} - 60227346872\,v^{78}z^{36} -$

$481393652408\,v^{76}z^{38} - 3377479989824\,v^{74}z^{40} - 19802866339002\,v^{72}z^{42} - 91156856020120\,v^{70}z^{44} -$

$303788277526368\,v^{68}z^{46} - 644839747658248\,v^{66}z^{48} - 650411067578792\,v^{64}z^{50} + 3738\,v^{88}z^{24} -$

$81949\,v^{86}z^{26} - 2489724\,v^{84}z^{28} - 35320329\,v^{82}z^{30} - 411968692\,v^{80}z^{32} -$

$4246482672\,v^{78}z^{34} - 39257042596\,v^{76}z^{36} - 322875201080\,v^{74}z^{38} - 2295519074094\,v^{72}z^{40} -$

$13487758309398\,v^{70}z^{42} - 61798428614368\,v^{68}z^{44} - 204293299492488\,v^{66}z^{46} -$

$429613835395952\,v^{64}z^{48} - 429253192698902\,v^{62}z^{50} - 3738\,v^{86}z^{24} - 81949\,v^{84}z^{26} -$

$1190676\,v^{82}z^{28} - 17782605\,v^{80}z^{30} - 228553420\,v^{78}z^{32} - 2532624240\,v^{76}z^{34} -$

$24631386348\,v^{74}z^{36} - 209143175576\,v^{72}z^{38} - 1511443533042\,v^{70}z^{40} - 8926566284406\,v^{68}z^{42} -$

$40825606690336\,v^{66}z^{44} - 134218857667176\,v^{64}z^{46} - 280247994591504\,v^{62}z^{48} - 277903364080166\,v^{60}z^{50} +$

$8\,v^{84}z^{24} - 6805\,v^{82}z^{26} - 419224\,v^{80}z^{28} - 8317855\,v^{78}z^{30} - 120118936\,v^{76}z^{32} -$

$1443953040\,v^{74}z^{34} - 14856135976\,v^{72}z^{36} - 130730747384\,v^{70}z^{38} - 963424221320\,v^{68}z^{40} -$

$5736385138534\,v^{66}z^{42} - 26261313190920\,v^{64}z^{44} - 86081446767648\,v^{62}z^{46} - 178860971045184\,v^{60}z^{48} -$

$176367077976718\,v^{58}z^{50} - 8\,v^{82}z^{24} - 6805\,v^{80}z^{26} - 175368\,v^{78}z^{28} -$

$3616979\,v^{76}z^{30} - 59528392\,v^{74}z^{32} - 784997904\,v^{72}z^{34} - 8599236920\,v^{70}z^{36} -$

$78752328728\,v^{68}z^{38} - 593799516408\,v^{66}z^{40} - 3575385400454\,v^{64}z^{42} - 16431798968088\,v^{62}z^{44} -$

$53843305980576\,v^{60}z^{46} - 111588492445632\,v^{58}z^{48} - 109632107846398\,v^{56}z^{50} - 97\,v^{78}z^{26} -$

$40644\,v^{76}z^{28} - 1420860\,v^{74}z^{30} - 27700888\,v^{72}z^{32} - 405801288\,v^{70}z^{34} -$

$4766342856\,v^{68}z^{36} - 45630131084\,v^{66}z^{38} - 353287767132\,v^{64}z^{40} - 2158304577150\,v^{62}z^{42} -$

$9988397518104\,v^{60}z^{44} - 32809642811748\,v^{58}z^{46} - 67987236890736\,v^{56}z^{48} - 66691637015470\,v^{54}z^{50} -$

$97\,v^{76}z^{26} - 15052\,v^{74}z^{28} - 520364\,v^{72}z^{30} - 12040648\,v^{70}z^{32} -$

$198764680\,v^{68}z^{34} - 2521728088\,v^{66}z^{36} - 25362022748\,v^{64}z^{38} - 202462884708\,v^{62}z^{40} -$

$1259632386014\,v^{60}z^{42} - 5889948133896\,v^{58}z^{44} - 19452439109684\,v^{56}z^{46} - 40407709421840\,v^{54}z^{48} -$

$39664131992126\,v^{52}z^{50} - v^{74}z^{26} - 1704\,v^{72}z^{28} - 162316\,v^{70}z^{30} - 4861384\,v^{68}z^{32} -$

$91754136\,v^{66}z^{34} - 1267957592\,v^{64}z^{36} - 13476869108\,v^{62}z^{38} - 111468995088\,v^{60}z^{40} -$

$709285560886\,v^{58}z^{42} - 3363598176840\,v^{56}z^{44} - 11205690532740\,v^{54}z^{46} - 23399408777656\,v^{52}z^{48} -$

$23038714793966\,v^{50}z^{50} - v^{72}z^{26} - 600\,v^{70}z^{28} - 49500\,v^{68}z^{30} -$

$1805848\, v^{66} z^{32} - 39613656\, v^{64} z^{34} - 602559432\, v^{62} z^{36} - 6818614148\, v^{60} z^{38} -$
$58780198512\, v^{58} z^{40} - 384435434454\, v^{56} z^{42} - 1856758637016\, v^{54} z^{44} - 6262056154548\, v^{52} z^{46} -$
$13184761205640\, v^{50} z^{48} - 13054246802462\, v^{48} z^{50} - 36\, v^{68} z^{28} - 11110\, v^{66} z^{30} -$
$605682\, v^{64} z^{32} - 15841881\, v^{62} z^{34} - 268819012\, v^{60} z^{36} - 3269192212\, v^{58} z^{38} -$
$29584744206\, v^{56} z^{40} - 200032884369\, v^{54} z^{42} - 988674278916\, v^{52} z^{44} - 3388904331554\, v^{50} z^{46} -$
$7218275465284\, v^{48} z^{48} - 7206654993255\, v^{46} z^{50} - 12\, v^{66} z^{28} - 2750\, v^{64} z^{30} -$
$181566\, v^{62} z^{32} - 5795361\, v^{60} z^{34} - 111686828\, v^{58} z^{36} - 1477229764\, v^{56} z^{38} -$
$14156533074\, v^{54} z^{40} - 99624501921\, v^{52} z^{42} - 506619547020\, v^{50} z^{44} - 1772707429386\, v^{48} z^{46} -$
$3833486268572\, v^{46} z^{48} - 3870883473423\, v^{44} z^{50} - 330\, v^{62} z^{30} - 46200\, v^{60} z^{32} - 1909413\, v^{58} z^{34} -$
$42805224\, v^{56} z^{36} - 625136356\, v^{54} z^{38} - 6411427800\, v^{52} z^{40} - 47333141361\, v^{50} z^{42} -$
$249176293440\, v^{48} z^{44} - 894399492630\, v^{46} z^{46} - 1971467062152\, v^{44} z^{48} - 2019911029035\, v^{42} z^{50} -$
$66\, v^{60} z^{30} - 10120\, v^{58} z^{32} - 555357\, v^{56} z^{34} - 14959320\, v^{54} z^{36} -$
$245914484\, v^{52} z^{38} - 2734111208\, v^{50} z^{40} - 21371954465\, v^{48} z^{42} - 117286813536\, v^{46} z^{44} -$
$434228377598\, v^{44} z^{46} - 979886699128\, v^{42} z^{48} - 1022325547011\, v^{40} z^{50} - 1540\, v^{56} z^{32} -$
$138699\, v^{54} z^{34} - 4697220\, v^{52} z^{36} - 89114636\, v^{50} z^{38} - 1091167476\, v^{48} z^{40} -$
$9130378717\, v^{46} z^{42} - 52656681168\, v^{44} z^{44} - 202323056340\, v^{42} z^{46} - 469693212092\, v^{40} z^{48} -$
$500954602365\, v^{38} z^{50} - 220\, v^{54} z^{32} - 28611\, v^{52} z^{34} - 1299276\, v^{50} z^{36} -$
$29411996\, v^{48} z^{38} - 404549772\, v^{46} z^{40} - 3671508333\, v^{44} z^{42} - 22461572496\, v^{42} z^{44} -$
$90199961124\, v^{40} z^{46} - 216597857700\, v^{38} z^{48} - 237188470869\, v^{36} z^{50} - 4455\, v^{50} z^{34} -$
$307824\, v^{48} z^{36} - 8709636\, v^{46} z^{38} - 138057800\, v^{44} z^{40} - 1381028269\, v^{42} z^{42} -$
$9062415408\, v^{40} z^{44} - 38344481652\, v^{38} z^{46} - 95831928248\, v^{36} z^{48} - 108270589665\, v^{34} z^{50} -$
$495\, v^{48} z^{34} - 59664\, v^{46} z^{36} - 2266644\, v^{44} z^{38} - 42856440\, v^{42} z^{40} -$
$482210141\, v^{40} z^{42} - 3439768752\, v^{38} z^{44} - 15480939684\, v^{36} z^{46} - 40554004360\, v^{34} z^{48} -$
$47530051593\, v^{32} z^{50} - 8712\, v^{44} z^{36} - 502788\, v^{42} z^{38} - 11911506\, v^{40} z^{40} -$
$154796067\, v^{38} z^{42} - 1220316192\, v^{36} z^{44} - 5908117356\, v^{34} z^{46} - 16355931672\, v^{32} z^{48} -$
$20009970690\, v^{30} z^{50} - 792\, v^{42} z^{36} - 90420\, v^{40} z^{38} - 2899342\, v^{38} z^{40} -$
$45118419\, v^{36} z^{42} - 401415040\, v^{34} z^{44} - 2119475932\, v^{32} z^{46} - 6260892328\, v^{30} z^{48} -$
$8052961842\, v^{28} z^{50} - 12012\, v^{38} z^{38} - 597960\, v^{36} z^{40} - 11742291\, v^{34} z^{42} -$
$121201456\, v^{32} z^{44} - 709898988\, v^{30} z^{46} - 2263623752\, v^{28} z^{48} - 3086739330\, v^{26} z^{50} -$
$924\, v^{36} z^{38} - 99000\, v^{34} z^{40} - 2665443\, v^{32} z^{42} - 33154096\, v^{30} z^{44} -$
$220157436\, v^{28} z^{46} - 768538488\, v^{26} z^{48} - 1122090258\, v^{24} z^{50} - 11880\, v^{32} z^{40} -$
$509575\, v^{30} z^{42} - 8073784\, v^{28} z^{44} - 62557924\, v^{26} z^{46} - 243323072\, v^{24} z^{48} -$

$384916410\, v^{22} z^{50} - 792\, v^{30} z^{40} - 77495\, v^{28} z^{42} - 1707976\, v^{26} z^{44} - 16067700\, v^{24} z^{46} -$
$71223232\, v^{22} z^{48} - 123860586\, v^{20} z^{50} - 8415\, v^{26} z^{42} - 302632\, v^{24} z^{44} - 3663324\, v^{22} z^{46} -$
$19067184\, v^{20} z^{48} - 37120290\, v^{18} z^{50} - 495\, v^{24} z^{42} - 42328\, v^{22} z^{44} - 722860\, v^{20} z^{46} -$
$4603856\, v^{18} z^{48} - 10270066\, v^{16} z^{50} - 4180\, v^{20} z^{44} - 118902\, v^{18} z^{46} - 984124\, v^{16} z^{48} -$
$2594275\, v^{14} z^{50} - 220\, v^{18} z^{44} - 15342\, v^{16} z^{46} - 181476\, v^{14} z^{48} - 589867\, v^{12} z^{50} -$
$1386\, v^{14} z^{46} - 27784\, v^{12} z^{48} - 118455\, v^{10} z^{50} - 66\, v^{12} z^{46} - 3320\, v^{10} z^{48} -$
$20463\, v^{8} z^{50} - 276\, v^{8} z^{48} - 2925\, v^{6} z^{50} - 12\, v^{6} z^{48} - 325\, v^{4} z^{50} - 25\, v^{2} z^{50} - z^{50})$
